# Pretreatment Thoracic CT Radiomic Features to Predict Brain Metastases in Patients With *ALK*-Rearranged Non-Small Cell Lung Cancer

**DOI:** 10.3389/fgene.2022.772090

**Published:** 2022-02-25

**Authors:** Hua Wang, Yong-Zi Chen, Wan-Hu Li, Ying Han, Qi Li, Zhaoxiang Ye

**Affiliations:** ^1^ Department of Radiology, Key Laboratory of Cancer Prevention and Therapy, Tianjin Medical University Cancer Institute and Hospital, National Clinical Research Center for Cancer, Tianjin Clinical Research Center for Cancer, Tianjin, China; ^2^ Laboratory of Tumor Cell Biology, Key Laboratory of Cancer Prevention and Therapy, Tianjin Clinical Research Center for Cancer, National Clinical Research Center for Cancer, Tianjin Medical University Cancer Institute and Hospital, Tianjin Medical University, Tianjin, China; ^3^ Department of Radiology, Shandong Cancer Hospital and Institute, Shandong First Medical University and Shandong Academy of Medical Sciences, Jinan, China; ^4^ Department of Biotherapy, Key Laboratory of Cancer Prevention and Therapy, Tianjin Medical University Cancer Institute and Hospital, National Clinical Research Center for Cancer, Tianjin Clinical Research Center for Cancer, Tianjin, China; ^5^ Department of Pathology, Key Laboratory of Cancer Prevention and Therapy, Tianjin Medical University Cancer Institute and Hospital, National Clinical Research Center for Cancer, Tianjin Clinical Research Center for Cancer, Tianjin, China

**Keywords:** radiomics, computed tomography, anaplastic lymphoma kinase, lung cancer, brain metastases

## Abstract

**Objective:** To identify CT imaging biomarkers based on radiomic features for predicting brain metastases (BM) in patients with *ALK*-rearranged non-small cell lung cancer (NSCLC).

**Methods:** NSCLC patients with pathologically confirmed *ALK* rearrangement from January 2014 to December 2020 in our hospital were enrolled retrospectively in this study. Finally, 77 patients were included according to the inclusion and exclusion criteria. Patients were divided into two groups: BM+ were those patients who were diagnosed with BM at baseline examination (*n* = 16) or within 1 year’s follow-up (*n* = 14), and BM− were those without BM followed up for at least 1 year (*n* = 47). Radiomic features were extracted from the pretreatment thoracic CT images. Sequential univariate logistic regression, LASSO regression, and backward stepwise logistic regression were used to select radiomic features and develop a BM-predicting model.

**Results:** Five robust radiomic features were found to be independent predictors of BM. AUC for radiomics model was 0.828 (95% CI: 0.736–0.921), and when combined with clinical features, the AUC was increased (*p* = 0.017) to 0.909 (95% CI: 0.845–0.972). The individualized BM-predicting model incorporated with clinical features was visualized by the nomogram.

**Conclusion:** Radiomic features extracted from pretreatment thoracic CT images have the potential to predict BM within 1 year after detection of the primary tumor in patients with *ALK*-rearranged NSCLC. The radiomics model incorporated with clinical features shows improved risk stratification for such patients.

## Introduction

Lung cancer is the leading cause of cancer-related mortality worldwide. Non-small cell lung cancer (NSCLC) accounts for 85% of all lung cancer incidence (Molina et al., 2008). Approximately 10%–20% of NSCLC patients have brain metastases (BMs) at initial presentation (Schuette, 2004; Khalifa et al., 2016). Another 25%–50% will develop BMs during the course of their disease (Langer and Mehta, 2005). It has been reported that 91% of BMs were diagnosed within 1 year of initial diagnosis of the primary tumor for patients with lung cancer (Schouten et al., 2002). For stage I–III NSCLC patients, the median time from treatment to onset of BMs as the first site of progression was 12 months (Bajard et al., 2004). NSCLC patients with BMs traditionally have a poor prognosis with a median survival of 7 months (Sperduto et al., 2010).

Anaplastic lymphoma kinase (*ALK*) rearrangements are driver mutations seen in about 3%–5% NSCLC (Gainor et al., 2013). The incidence of BMs is higher in patients with *ALK*-rearranged NSCLC: among those patients, up to 50%–60% will develop BMs during the course of their disease (Zhang et al., 2015). Crizotinib was the first *ALK* inhibitor developed and has demonstrated improved outcomes in patients with *ALK*-positive advanced NSCLC in comparison with chemotherapy (Solomon et al., 2014). However, the intracranial efficacy of crizotinib is poor, due to poor blood–brain barrier penetration (Costa et al., 2011). Second- and third-generation *ALK* inhibitors have shown better but variable intracranial control. Besides, prophylactic cranial irradiation has been discussed as a strategy to reduce the incidence of BM in NSCLC (Carolan et al., 2005; Pechoux et al., 2016). Therefore, developing biomarkers to predict patients at higher risk of BM might be significant in helping identify sub-groups who need early detection of BM by close observation and benefit from intensification of systemic therapy, which is crucial for improving outcomes.

Tumor phenotypic differences can be quantified in CT images using radiomic features. Radiomics refers to high-throughput extraction of quantitative image features, which provide a comprehensive description of tumor phenotypes and heterogeneity (Kumar et al., 2012; Lambin et al., 2012). Biomarkers based on radiomic features have been reported to be associated with clinical outcomes and underlying genomic patterns (Chen et al., 2017). In recent years, studies have been performed on the predictive value of radiomic features for tumor progression and distant metastases in NSCLC (Fried et al., 2014; Coroller et al., 2015; Fan et al., 2019; Xu et al., 2019; Kakino et al., 2020; Sun et al., 2021). However, to date, research using a radiomics approach based on thoracic CT images to predict BM for *ALK*-rearranged NSCLC has been rarely reported (Xu et al., 2019). The purpose of this study was to identify CT imaging biomarkers using radiomic features extracted from pretreatment thoracic CT images for predicting BM in patients with *ALK*-rearranged NSCLC, focused on BM within 1 year after initial detection of the primary tumor.

## Materials and Methods

### Study Population and Clinical Data

NSCLC patients with pathologically confirmed *ALK* rearrangement from January 2014 to December 2020 in our hospital were enrolled retrospectively in this study. Patients were consecutively included according to the following inclusion criteria: (1) pathologically confirmed NSCLC with *ALK* rearrangement; (2) available pretreatment thoracic CT images on picture archiving and communication system (PACS) performed less than 1 month before the pathologic sampling were collected; and (3) available brain MRI/PETCT/CT examination data at diagnosis of NSCLC and during follow-up to confirm the status of BMs. Patients who met any of the following criteria were excluded: (1) with other malignant neoplasms; (2) unsatisfactory CT image quality such as severe respiratory motion artifacts; and (3) loss to follow-up within 1 year and without BM at the last follow-up.

**FIGURE 1:**
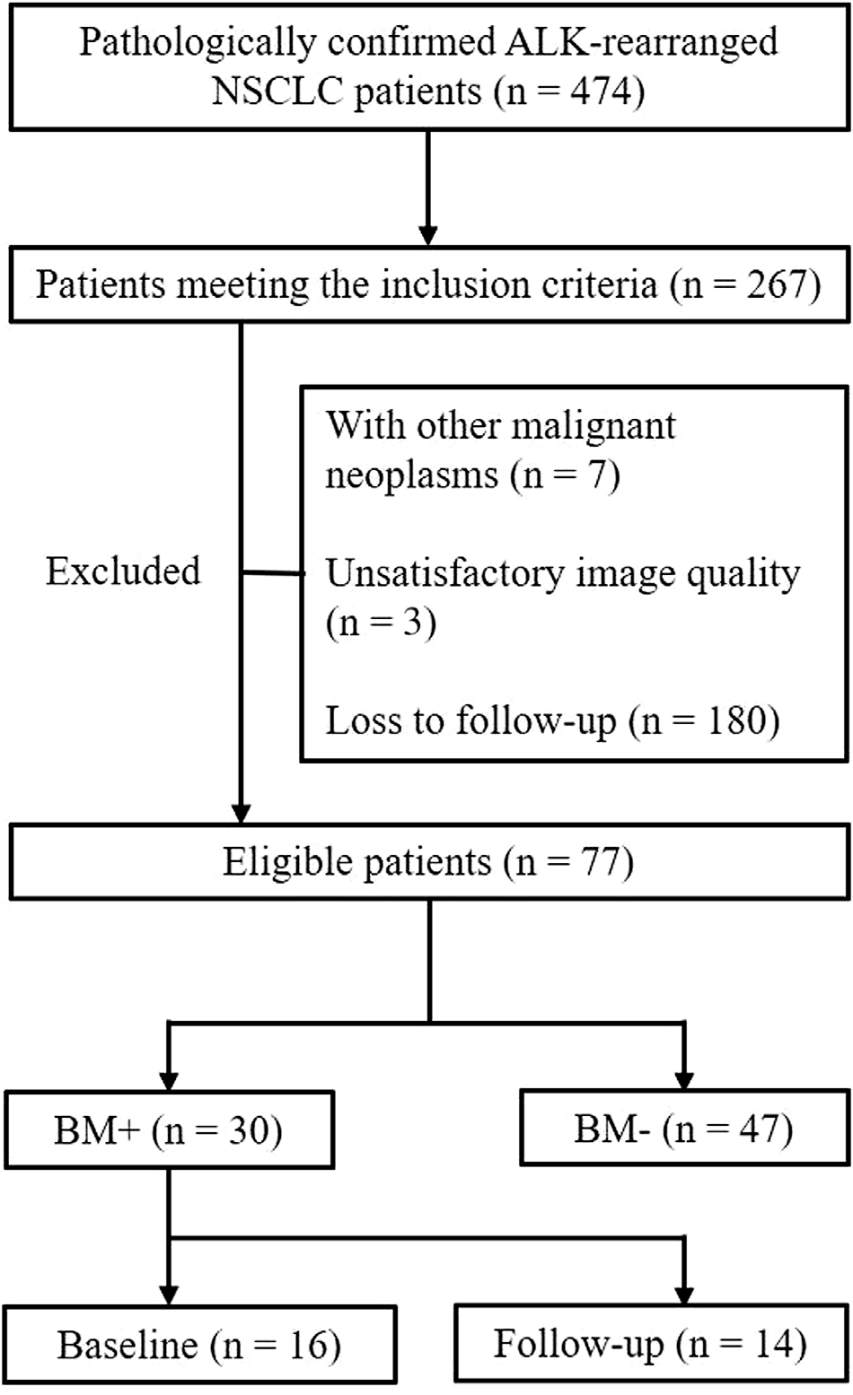
Flowchart of the patient selection process.

Finally, 77 patients were included in the study. Patients were divided into two groups: BM+ were those patients who diagnosed BM at baseline examination (*n* = 16) or within 1 year’s follow-up (*n* = 14), and BM− were those without BM followed up for at least 1 year (*n* = 47) (Figure 1).

Clinicopathologic features were extracted from patient medical records, including age at diagnosis, sex, smoking status, pathological type, and TNM stage. Tumors were staged according to the new eighth edition of the Union for International Cancer Control and American Joint Committee on Cancer TNM classification system (Detterbeck et al., 2017).

### CT Acquisition, Image Segmentation, and Feature Extraction

Pretreatment chest CT examinations were performed using one of the three multi-detector CT systems: Somatom Definition AS+ (Siemens Medical Solutions), Light speed 16 (GE Healthcare), or Discovery CT750 HD (GE Healthcare) scanner. Scanning parameters were as follows: tube voltage, 120 kVp; tube current, 150–200 mA with automatic exposure control; reconstruction thicknesses and intervals were 1.5 mm or 1.25 mm; reconstruction kernel was B30f/Standard for mediastinal window, and B70f/Lung for lung window.

The tumors were segmented using a semi-automatic approach by one radiologist and reviewed by another one, both of whom had experience in thoracic CT diagnosis for more than 10 years. They were both blinded to the clinical data and pathologic information except for lung cancer diagnosis. 3D Slicer V4.11.0^1^ (Fedorov et al., 2012), an open-source image processing software, was used to segment the tumors on the images with reconstruction kernel of B70f/Lung and extract three-dimensional (3D) Radiomic features.

Features are grouped as follows: (1) First-order features: These describe the voxel intensity distribution in the delineated ROI. They are usually calculated based on the intensity histogram, including energy, entropy, skewness, kurtosis, uniformity, mean, minimum, and maximum intensity values. (2) Shape features: descriptors of the two- and three-dimensional shape and size of the ROI. (3) Textural features: These contain gray-level co-occurrence matrix (GLCM), gray-level dependence matrix (GLDM), gray-level run length matrix (GLRLM), gray-level size zone matrix (GLSZM), and neighborhood gray tone difference matrix (NGTDM). They are computed on the analysis of the three-dimensional directions within the tumor and the consideration of the spatial location of each voxel in the ROI (Shafiq-Ul-Hassan et al., 2017; Xu et al., 2020). (4) Wavelet-based features: These are extracted after applying a series of wavelet filtration to the images. The wavelet transform decomposes the original image into low-and high-frequencies, focusing the features on different frequency ranges within the tumor volume (Rios Velazquez et al., 2017). Finally, a total of 851 features were extracted, including 14 shape features, 18 first-order features, 75 texture features (24 GLCM, 14 GLDM, 16 GLRLM, 16 GLSZM, and 5 NGTDM), and 744 wavelet-based features (Supplementary Table S1).

### Feature Selection, Radiomic Signature Building, and Development of Prediction Model

Univariate logistic regression analysis was preliminarily used to screen and identify potential predictors from radiomic features. Then, radiomic features with *p* < 0.05 in univariate analysis were further screened by the least absolute shrinkage and selection operator (LASSO) regression method. Tenfold cross-validation was used for selecting features in the LASSO model *via* minimum criteria. In addition, multivariate logistic regression using a backward elimination strategy was performed to eliminate the redundant features. Finally, the prediction model was established based on the simplified radiomic features with beta values included in the backward stepwise regression as the standardized regression coefficients. A radiomics score (Rad_score) was calculated for each patient *via* a linear combination of selected features weighted by their regression coefficients. To provide the clinician with a quantitative tool to predict the individual probability of BM within 1 year after detection of NSCLC, we also built a nomogram incorporated with clinical features.

### Statistical Analyses

Statistical analyses were conducted by R software (V3.6.2)^2^. For the potential clinical prognostic factors, the Student’s *t*-test was used to compare the age of the two groups, and the other clinical features were compared using chi-square or Fisher’s exact test, where appropriate. The diagnostic efficacy of the clinical, radiomic, and the combined model were analyzed by the receiver operating characteristic (ROC) curve of the subjects, and the differences between the area under the curve (AUC) were compared using DeLong’s test. All tests were two-sided. A *p*-value < 0.05 was defined as significant for all the tests, except that in multivariate logistic regression with backward elimination strategy, a *p*-value < 0.1 was considered significant so that potential predictors were less likely to be eliminated from the prediction model.

## Results

### Clinical Features

The patients’ clinical data are presented in Table 1. There were significant differences in T stage (*p* = 0.001) and N stage (*p* < 0.001) between the two groups. Those patients with a higher T or N stage tend to have BM within 1 year after detection of NSCLC.

**TABLE 1:** Demographic and clinical features of the patients.

Clinical features	BM+	BM−	Total	*p*-value
Age, mean ± SD, years	52.23 ± 12.85	55.68 ± 9.19	54.34 ± 10.81	0.208
Age distribution				0.743
≤60	20 (66.7)	33 (70.2)	53 (68.8)	
>60	10 (33.3)	14 (29.8)	24 (31.2)	
Sex, *N* (%)				0.522
Female	15 (50.0)	27 (57.4)	42 (54.5)	
Male	15 (50.0)	20 (42.6)	35 (45.5)	
Smoking status, *N* (%)				0.217
Never	22 (73.3)	28 (59.6)	50 (64.9)	
Ever	8 (26.7)	19 (40.4)	27 (35.1)	
Pathology, *N* (%)				0.140
Adenocarcinoma	29 (96.7)	40 (85.1)	69 (89.6)	
Other	1 (3.3)	7 (14.9)	8 (10.4)	
T stage, *N* (%)				**0.001**
T1/T2	12 (40.0)	37 (78.7)	49 (63.6)	
T3/T4	18 (60.0)	10 (21.3)	28 (36.4)	
N stage, *N* (%)				**<0.001**
N0/N1	3 (10.0)	27 (57.4)	30 (39.0)	
N2/N3	27 (90.0)	20 (42.6)	47 (61.0)	

Abbreviations: BM, brain metastases.

Bolded values indicate a statistically significant result.

### Radiomic Signature Building

**FIGURE 2:**
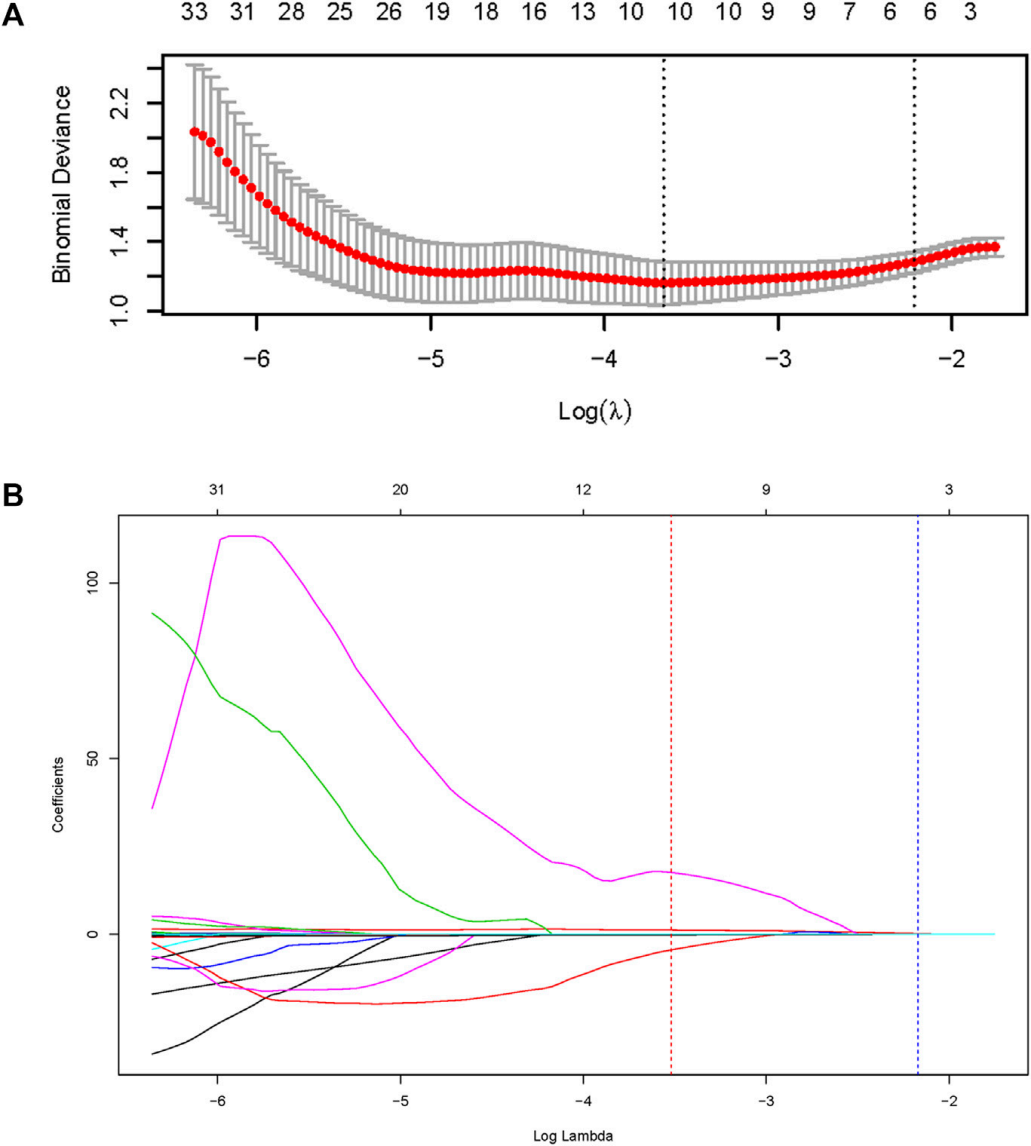
Feature selection using the least absolute shrinkage and selection operator (LASSO) regression method. **(A)** The dotted vertical line was plotted at the value selected by the 10-fold cross-validation *via* minimum criteria (the value of lambda with the lowest partial likelihood deviance). **(B)** Selection of the tuning parameter (lambda) in the LASSO regression using 10-fold cross-validation *via* minimum criteria.

Radiomic signature was built *via* three sequential steps. Firstly, a total of 112 radiomic features associated with BM (*p* < 0.05) were preliminarily identified by univariate logistic regression analysis (Supplementary Table S2). Then, ten radiomic features remained after conducting LASSO regression (Figure 2). Eventually, five robust radiomic features were found to be independent predictors of BM by using a backward stepwise logistic regression (Table 2). A detailed description of the features is presented in Supplementary S1. The prediction model based on the five radiomic features was built, and Rad_score was calculated for each patient. The Rad_score calculation formula was as follows:

**TABLE 2:** Multivariate logistic regression analyses of radiomic features.

Radiomic features	Beta value	Odds ratio (95% CI)	*p-*value	AUC
Original.GLCM.contrast	−0.027	0.973 (0.942–1.006)	0.109	0.600
Wavelet_LHH.GLCM.clusterShade	0.046	1.047 (1.012–1.083)	0.009	0.666
Wavelet_LLH.GLSZM.smallAreaEmphasis	−30.675	0 (0.000–0.045)	0.014	0.632
Wavelet_HLH.firstorder.maximum	0.004	1.004 (1.000–1.007)	0.071	0.657
Wavelet_LLL.firstorder.skewness	−0.355	0.701 (0.498–0.985)	0.041	0.656

Abbreviations: CI, confidence interval; AUC, area under the receiver operating characteristic curve.

Rad_score = Wavelet_LHH.GLCM.ClusterShade * 0.0459−Original. GLCM.Contrast * 0.0270−Wavelet_LLH.GLSZM.SmallAreaEmphasis * 3.6752 + Wavelet_HLH.Firstorder.Maximum * 0.0036−Wavelet_LLL.Firstorder.Skewness * 0.3551.

### Development of an Individualized Prediction Model

**FIGURE 3:**
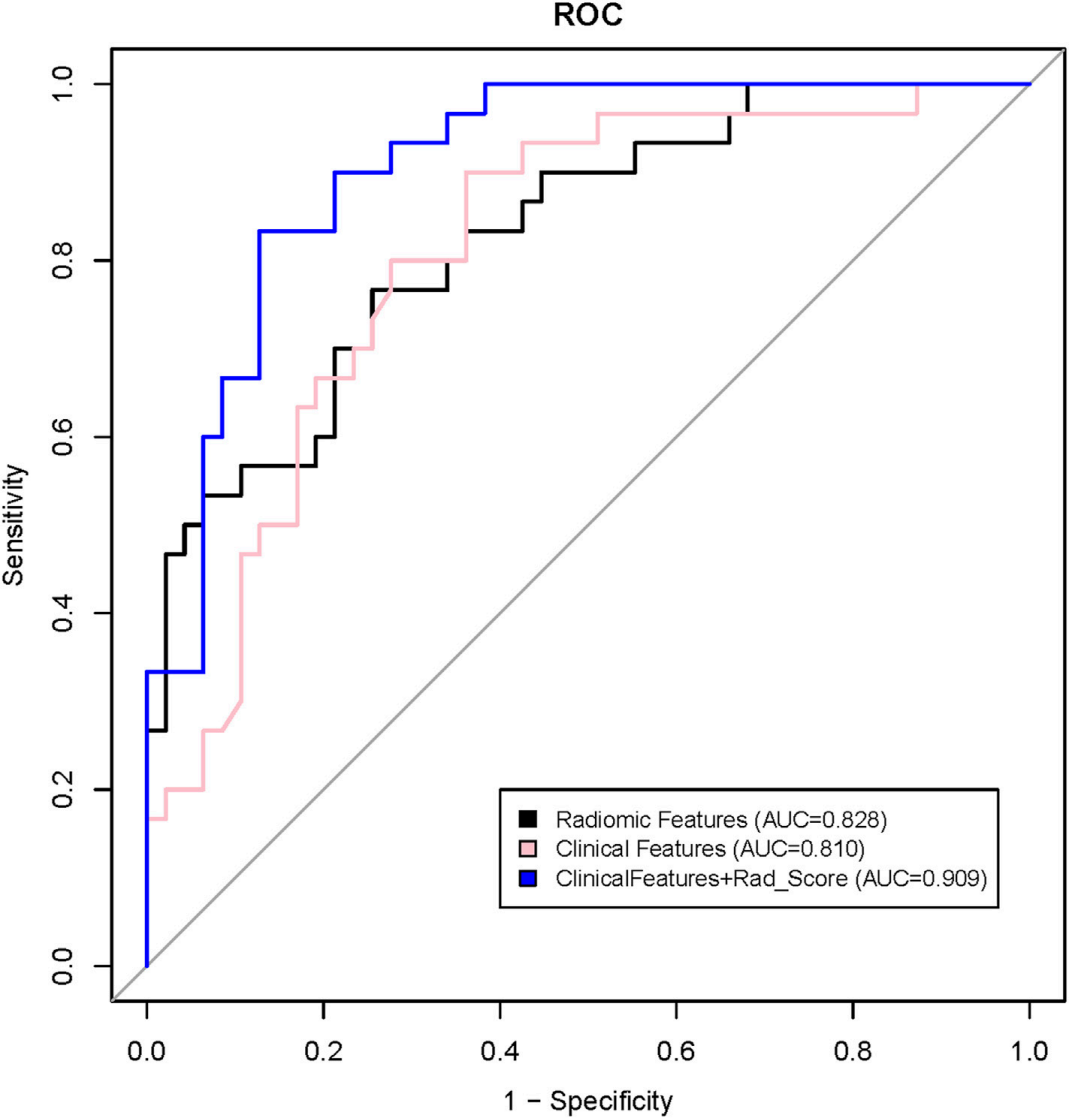
Receiver operating characteristic (ROC) curves for prediction of brain metastases using a clinical model (pink line), a radiomic model (black line), and a model that combined Radiomics score (Rad_score) and clinical features (blue line).

**FIGURE 4:**
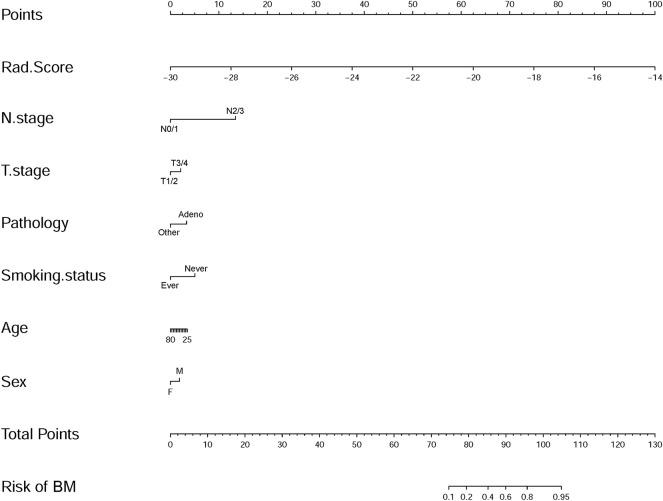
Nomogram developed with the radiomics score (Rad_score) and clinical features incorporated.

**FIGURE 5:**
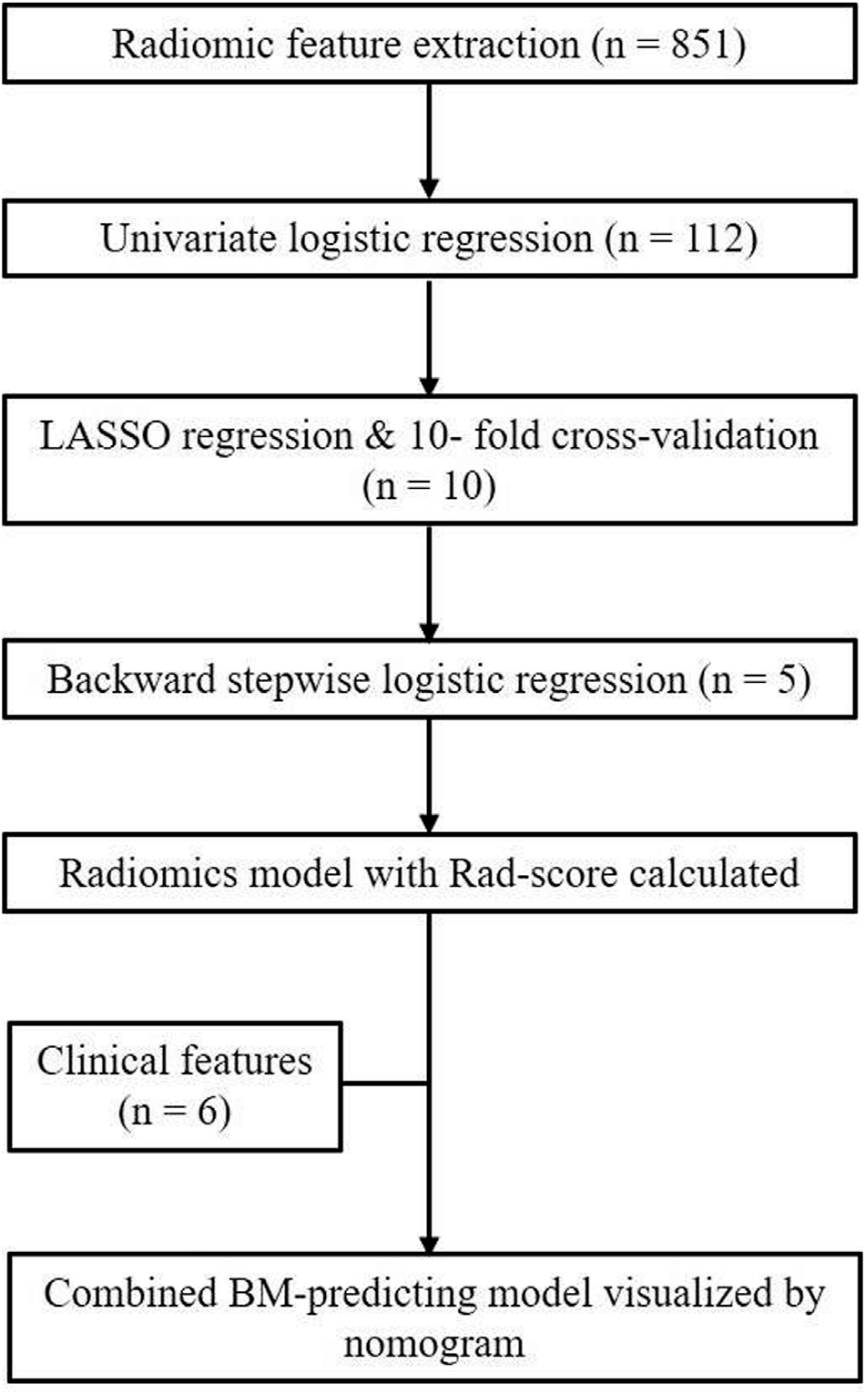
Flowchart of the process involved in the development of the prediction model.

To illustrate the potential ability for BM prediction, we compared the models developed by radiomic features, clinical variables, and a combination of them. As shown in Figure 3, AUC for the radiomics model was 0.828 (95% CI: 0.736–0.921), which showed no significant difference (*p* = 0.785) with the clinical model (AUC = 0.810, 95% CI: 0.712–0.908), and when combined with clinical features, the AUC of the radiomics model was increased (*p* = 0.017) to 0.909 (95% CI: 0.845–0.972). The combined model was also superior to the clinical model alone (*p* = 0.028). The individualized BM-predicting model incorporated with clinical features is visualized by the nomogram (Figure 4). The process involved in the development of the prediction model is shown with a flowchart (Figure 5).

## Discussion

In this study, we developed a radiomics model with five independent predictors out of 851 candidate radiomic features extracted from pretreatment thoracic CT images for predicting BM within 1 year after detection of the primary tumor in patients with *ALK*-rearranged NSCLC, which showed a good performance with an AUC of 0.828. Furthermore, incorporating the radiomics signature with clinical features resulted in a significant improvement of predictive power with an excellent model performance (AUC = 0.909). We also built an easy-to-use nomogram that facilitates the individualized prediction of BM.

Age, T/N stage, pathological type, tumor genes, and other clinical features have been reported as risk factors or potential predictors of BMs. Patients with younger age (≤60 years), later T/N stage, adenocarcinoma, or non-squamous NSCLC are associated with a higher risk of BM (Robnett et al., 2001; Bajard et al., 2004; Carolan et al., 2005; Shi et al., 2006; Ji et al., 2014; Won et al., 2015). Epidermal growth factor receptor (*EGFR*) mutation was also reported to be a potential risk factor of BM (Shin et al., 2014; Shin et al., 2016). Compared with *EGFR* mutant patients, BMs were more common in patients with *ALK* rearrangement (Kang et al., 2014). Published data on risk factors of BM concerning the clinical features of *ALK*-rearranged NSCLC are minimal (Costa et al., 2015; Johung et al., 2016). Patients of this molecular subtype of NSCLC are relatively young (Yamamoto et al., 2014). While Costa et al. found younger age was associated with BM (Costa et al., 2015), no significant association between age and BM was found by Johung et al. (2016). In our study, most of the patients were younger than 60 years (68.8%); though patients in the BM+ group appeared younger than those in the BM− group (52.23 vs. 55.68 years), no significant difference was presented. Like previous studies (Bajard et al., 2004; Ji et al., 2014; Won et al., 2015), we also found that later T/N stage was associated with a higher risk of BM. Though up to 96.7% of patients in BM+ group were adenocarcinoma, no significant association was found between pathological type and BM. It might due to the high prevalence of adenocarcinoma (89.6%) in this cohort, which is consistent with a previous report where adenocarcinoma accounts for most cases (85.3%) of *ALK*-rearranged NSCLC (Barlesi et al., 2016).

On account of the limited value of the clinical prognostic factors in predicting BM in this specific patient subset with a high incidence of BM, developing other biomarkers to build an optimal prediction model is necessary. Radiomics, as a non-invasive method developed in recent years, may potentially improve predictive accuracy in oncology. We found that five radiomic features, including one texture feature (Original.GLCM.Contrast), two wavelet-transformed texture features (Wavelet_LHH.GLCM.ClusterShade and Wavelet_LLH.GLSZM.SmallAreaEmphasis), and two wavelet-transformed first-order features (Wavelet_HLH.Firstorder.Maximum and Wavelet_LLL.Firstorder.Skewness), were independent predictors of BM in patients with *ALK*-rearranged NSCLC. The radiomics signature incorporated with clinical features yielded significantly improved predictive performance compared to both the radiomics model and the clinical model alone. Maximum and Skewness measure the maximal intensity of the histogram and the asymmetry of the histogram from the mean, respectively. Texture features are known to be most closely correlated with tumor heterogeneity and prognosis among all radiomic features, while wavelet-based features are the results of filter transformation of intensity and texture features (Chen et al., 2017). GLCM, ClusterShade, and Skewness (original or filtered) have been reported to be predictors of distant metastases in NSCLC (Coroller et al., 2015; Huynh et al., 2017; Kakino et al., 2020). Sun et al. (2021) also found that GLCM and GLSZM features were predictors of BM as the first failure in patients with curatively resected locally advanced NSCLC. Although differences exist in study objective and implementation, it implies that such features may serve as a risk factor of distant metastases, including BM for NSCLC. Further investigation is needed to explore the extensibility and universal applicability of these radiomic features for NSCLC with other driver gene mutations or distant metastases of other sites.

Recently, Xu et al. (2019) tried to build a radiomic signature to predict pretreatment BM for stage III/IV *ALK*-positive NSCLC patients and found that only one radiomic feature (W_GLCM_LH_Correlation) was an independent predictor (training set: AUC = 0.687, test set: AUC = 0.642), which also exhibited reposeful performance in predicting BM during follow-up (stage III: AUC = 0.682, stage IV: AUC = 0.653). However, due to the low positive rate (27 patients with pretreatment BM out of 132 patients) in their research, splitting data to the training set and test set and further dividing patients without BM at baseline examination into groups of different stages subsequently reduced sample size, which would mitigate statistical power compared to the initial cohort. To overcome this, we combined the patients with BM at baseline examination and within 1 year’s follow-up into the BM+ group. We then used a cross-validation approach, which employs repeated data-splitting to prevent overfitting while simultaneously generating estimates of the model coefficients. This process is almost equivalent to data-splitting in producing validated model coefficients. Still, its use of data is more efficient than a dichotomous split into training and test sets (Harrell et al., 1996). However, there remains a high risk of a false-positive result due to the multiplicity of testing with the number of features tested (Fried et al., 2014). Additionally, recent studies have revealed that BM can occur even in patients with early-stage NSCLC or in those without any symptoms (Shi et al., 2006; Ando et al., 2018). Therefore, we did not intentionally exclude the patients with early stage. Actually, in the BM+ group, 40% were T0/1 stage, and 10% were N1/2 stage at the initial diagnosis.

There are several limitations to this study. First, due to the low incidence of *ALK* rearrangement and the high proportion of loss to follow-up, the sample size of our study was relatively small. Therefore, we only performed internal cross-validation, and the independent model assessment could not be committed to avoid overfitting. Expanded sample size and external multicenter validation are necessary for further investigation to confirm our findings. Second, the CT acquisition and reconstruction parameters were not consistent for all the cases due to the different CT scanners we used. However, radiomics was able to detect a solid signal to predict BM despite the variability. In addition, because some patients did not undergo enhanced CT in the present study, we used plain CT images to extract the radiomic features to keep the sample size as large as possible, which may have an effect on the segmentation of the tumor.

In conclusion, our preliminary study indicates that radiomic features derived from pretreatment thoracic CT images may function as non-invasive biomarkers for predicting BM in patients with *ALK*-rearranged NSCLC. Furthermore, the radiomics model incorporated with clinical features shows improved risk stratification for such patients, allowing individualized treatment to reduce the risk of BM and improve survival.

## Data Availability

The original contributions presented in the study are included in the article/Supplementary Material, further inquiries can be directed to the corresponding author.

## Footnotes

1
http://www.slicer.org.

2
http://www.r-project.org.

## Abbreviations

ALK, anaplastic lymphoma kinase; AUC, area under the curve; BM, brain metastasis; GLCM, gray-level co-occurrence matrix; GLDM, gray-level dependence matrix; GLRLM, gray-level run length matrix; GLSZM, gray-level size zone matrix; LASSO, least absolute shrinkage and selection operator; NGTDM, neighborhood gray tone difference matrix; NSCLC, non-small cell lung cancer.
