# Supplementary material for: Pretreatment Thoracic CT Radiomic Features to Predict Brain Metastases in Patients With ALK-Rearranged Non-Small Cell Lung Cancer
Source: Front Genet. 2022 Feb 25;13:772090. doi: 10.3389/fgene.2022.772090 (PMC8914538; doi:10.3389/fgene.2022.772090)
Supplement: Supplementary file 1 [file DataSheet2.pdf]

Supplementary Table S2. Univariate logistic regression analysis for radiomic features

Radiomic Feature	Odds Ratio			p-value
	Point	95%CI		
		Lower	Upper	
F1	1.000	1.000	1.000	0.013
F2	1.008	0.997	1.019	0.173
F3	1.000	1.000	1.000	0.013
F4	1.012	0.995	1.032	0.190
F5	0.071	0.001	5.124	0.231
F6	1.048	1.007	1.095	0.028
F7	0.258	0.007	8.250	0.441
F8	0.213	0.019	1.110	0.155
F9	1.013	0.999	1.030	0.096
F10	0.428	0.021	8.509	0.571
F11	1.000	1.000	1.000	0.014
F12	1.036	1.004	1.073	0.031
F13	1.023	1.003	1.045	0.027
F14	1.010	0.995	1.027	0.199
F15	0.972	0.937	0.999	0.080
F16	1.000	1.000	1.001	0.298
F17	0.970	0.243	4.041	0.964
F18	1.000	1.000	1.000	0.024
F19	1.000	1.000	1.000	0.010
F20	0.014	0.000	1.028	0.070
F21	1.000	0.998	1.002	0.854
F22	0.001	0.000	1.175	0.092
F23	1.012	0.966	1.060	0.603
F24	0.677	0.208	1.227	0.317
F25	1.026	0.862	1.218	0.763
F26	1.000	1.000	1.000	0.462
F27	0.000	0.000	2.63E+07	0.205
F28	0.000	0.000	108.586	0.205
F29	1.021	0.979	1.068	0.333
F30	1.011	0.989	1.033	0.333
F31	0.841	0.524	1.330	0.461
F32	1.000	1.000	1.000	0.875
F33	0.492	0.000	5.20E+09	0.952
F34	1.69E+23	39.172	1.95E+50	0.058
F35	7.995	0.000	1.38E+23	0.937
F36	0.974	0.948	0.997	0.041
F37	0.526	0.186	1.414	0.210
F38	42.716	0.111	2.10E+04	0.221
F39	0.952	0.895	1.000	0.076
F40	1.03E+07	0.517	1.96E+15	0.074
F41	20.579	0.073	6.80E+03	0.295
F42	0.438	0.016	8.716	0.599
F43	1.000	1.000	1.001	0.281

F44	0.521	0.194	1.264	0.169
F45	0.520	0.027	8.567	0.652
F46	0.970	0.933	0.998	0.077
F47	1.000	1.000	1.000	0.513
F48	0.102	0.007	1.048	0.068
F49	2.60E+04	2.082	1.41E+11	0.123
F50	0.720	0.497	1.006	0.065
F51	45.043	0.085	3.01E+04	0.238
F52	0.991	0.977	1.000	0.112
F53	0.989	0.977	0.999	0.053
F54	0.768	0.503	1.096	0.184
F55	4.35E+03	0.002	1.48E+10	0.258
F56	1.029	1.005	1.062	0.048
F57	1.000	1.000	1.000	0.057
F58	0.974	0.945	0.997	0.050
F59	0.982	0.963	0.998	0.050
F60	1.000	1.000	1.000	0.051
F61	1.001	0.999	1.002	0.374
F62	0.990	0.977	0.999	0.113
F63	1.007	0.999	1.019	0.175
F64	1.000	0.998	1.002	0.864
F65	0.452	0.157	1.190	0.120
F66	1.000	0.999	1.001	0.442
F67	1.000	1.000	1.000	0.081
F68	1.009	1.002	1.019	0.044
F69	0.996	0.959	1.024	0.800
F70	1.022	1.004	1.048	0.062
F71	0.000	0.000	198.202	0.170
F72	0.970	0.933	0.998	0.072
F73	0.000	0.000	124.440	0.182
F74	3.08E+04	0.002	8.73E+11	0.221
F75	8.419	0.010	7.57E+03	0.529
F76	1.000	1.000	1.000	0.010
F77	2.539	0.185	36.131	0.481
F78	1.000	1.000	1.001	0.308
F79	1.000	1.000	1.000	0.016
F80	0.000	0.000	5.03E+03	0.348
F81	1.000	1.000	1.001	0.295
F82	0.004	0.000	649.710	0.362
F83	0.000	0.000	119.184	0.255
F84	0.295	0.059	1.212	0.110
F85	1.000	1.000	1.001	0.299
F86	0.016	0.000	36.445	0.298
F87	0.982	0.955	1.005	0.154
F88	1.000	1.000	1.000	0.169
F89	3.87E+08	0.000	6.80E+22	0.207
F90	0.000	0.000	0.380	0.074

F91	1.000	1.000	1.000	0.025
F92	1.001	1.000	1.002	0.020
F93	1.000	1.000	1.000	0.169
F94	1.000	0.999	1.001	0.363
F95	0.030	0.001	0.968	0.062
F96	1.026	0.985	1.083	0.264
F97	1.000	1.000	1.000	0.140
F98	1.000	1.000	1.001	0.298
F99	0.000	0.000	0.352	0.074
F100	0.000	0.000	4.45E+11	0.262
F101	1.043	0.329	3.444	0.942
F102	0.000	0.000	7.36E+13	0.288
F103	0.000	0.000	4.93E+03	0.351
F104	1.000	1.000	1.000	0.391
F105	0.628	0.354	0.934	0.066
F106	0.000	0.000	0.117	0.038
F107	1.426	1.081	1.989	0.021
F108	0.985	0.959	1.008	0.211
F109	1.000	1.000	1.000	0.184
F110	1.446	0.382	7.413	0.607
F111	1.000	1.000	1.000	0.026
F112	1.000	1.000	1.000	0.011
F113	0.022	0.000	1.149	0.078
F114	1.000	1.000	1.001	0.366
F115	0.003	0.000	1.270	0.105
F116	1.019	0.978	1.063	0.370
F117	72.327	0.000	2.99E+10	0.537
F118	1.039	0.910	1.186	0.562
F119	1.000	1.000	1.000	0.206
F120	0.000	0.000	8.14E+13	0.290
F121	0.000	0.000	2.17E+15	0.248
F122	1.021	0.995	1.053	0.137
F123	1.011	0.997	1.026	0.137
F124	0.841	0.518	1.344	0.469
F125	1.000	0.999	1.001	0.646
F126	49.715	0.000	1.40E+11	0.722
F127	2.62E+34	3.25E+04	5.84E+73	0.050
F128	1.34E+04	0.000	4.59E+24	0.687
F129	0.989	0.972	1.004	0.162
F130	0.561	0.194	1.537	0.267
F131	62.658	0.123	4.21E+04	0.199
F132	0.986	0.954	1.015	0.357
F133	2.23E+10	38.987	4.95E+20	0.032
F134	34.550	0.121	1.25E+04	0.224
F135	0.141	0.001	30.464	0.475
F136	1.000	1.000	1.000	0.183
F137	0.566	0.211	1.426	0.237

F138	1.619	0.043	60.759	0.792
F139	0.980	0.949	1.006	0.156
F140	1.000	1.000	1.000	0.323
F141	0.145	0.006	2.618	0.202
F142	40.202	0.201	1.18E+05	0.259
F143	0.786	0.569	1.053	0.122
F144	66.296	0.126	4.70E+04	0.195
F145	0.991	0.977	1.003	0.157
F146	0.992	0.981	1.001	0.101
F147	1.126	0.579	2.237	0.724
F148	5.26E+03	0.003	1.98E+10	0.246
F149	1.043	0.986	1.118	0.182
F150	1.000	1.000	1.000	0.046
F151	0.980	0.956	1.003	0.103
F152	0.988	0.971	1.003	0.127
F153	1.000	1.000	1.000	0.044
F154	1.001	0.999	1.002	0.267
F155	0.993	0.981	1.004	0.198
F156	0.991	0.980	1.002	0.118
F157	0.999	0.998	1.000	0.142
F158	0.521	0.189	1.349	0.188
F159	1.001	1.000	1.001	0.143
F160	1.000	1.000	1.000	0.213
F161	1.006	0.998	1.014	0.146
F162	1.038	0.978	1.107	0.227
F163	1.022	0.985	1.062	0.258
F164	0.000	0.000	4.44E+14	0.246
F165	0.986	0.961	1.008	0.223
F166	0.000	0.000	3.63E+15	0.250
F167	5.66E+04	0.003	3.21E+12	0.208
F168	14.554	0.049	5.59E+03	0.356
F169	1.000	1.000	1.000	0.011
F170	3.484	0.339	40.457	0.296
F171	1.000	1.000	1.000	0.187
F172	1.000	1.000	1.000	0.018
F173	0.000	0.000	224.475	0.203
F174	1.000	1.000	1.000	0.173
F175	0.001	0.000	79.029	0.231
F176	0.000	0.000	4.26E+20	0.300
F177	0.445	0.106	1.718	0.250
F178	1.000	1.000	1.000	0.183
F179	0.006	0.000	10.727	0.186
F180	0.996	0.980	1.012	0.623
F181	1.000	1.000	1.000	0.151
F182	1.56E+09	0.000	1.44E+25	0.243
F183	0.000	0.000	2.126	0.129
F184	1.000	1.000	1.000	0.032

F185	1.001	1.000	1.002	0.022
F186	1.000	1.000	1.000	0.151
F187	1.000	1.000	1.001	0.215
F188	0.039	0.001	1.087	0.070
F189	1.037	0.988	1.118	0.215
F190	1.000	1.000	1.000	0.064
F191	1.000	1.000	1.000	0.179
F192	0.000	0.000	3.312	0.115
F193	0.000	0.000	2.71E+17	0.290
F194	1.905	0.496	10.514	0.393
F195	0.000	0.000	7.80E+18	0.306
F196	0.000	0.000	7.85E+03	0.332
F197	1.000	1.000	1.000	0.254
F198	0.942	0.815	1.044	0.328
F199	0.007	0.000	1.244	0.148
F200	1.374	0.932	2.125	0.123
F201	0.987	0.964	1.007	0.232
F202	1.000	1.000	1.000	0.384
F203	1.397	0.349	8.194	0.655
F204	1.000	1.000	1.000	0.020
F205	1.000	1.000	1.001	0.010
F206	0.030	0.000	2.266	0.130
F207	1.000	0.999	1.001	0.705
F208	0.010	0.000	3.837	0.164
F209	1.034	0.948	1.130	0.443
F210	0.000	0.000	1.22E+07	0.596
F211	1.082	0.821	1.428	0.565
F212	1.000	1.000	1.000	0.483
F213	0.000	0.000	7.06E+17	0.340
F214	0.000	0.000	1.13E+12	0.382
F215	1.016	0.985	1.050	0.315
F216	1.008	0.992	1.025	0.315
F217	0.924	0.558	1.527	0.753
F218	0.999	0.998	1.001	0.351
F219	0.049	0.000	2.32E+16	0.886
F220	3.44E+20	6.847	2.28E+45	0.067
F221	0.000	0.000	4.46E+29	0.754
F222	0.994	0.982	1.003	0.236
F223	0.634	0.202	1.895	0.419
F224	65.891	0.017	3.29E+05	0.323
F225	0.986	0.959	1.008	0.246
F226	6.62E+07	2.038	1.89E+16	0.052
F227	40.348	0.012	1.69E+05	0.374
F228	1.357	0.011	183.376	0.901
F229	1.000	1.000	1.000	0.367
F230	0.640	0.218	1.795	0.400
F231	0.476	0.006	28.272	0.725

F232	0.986	0.962	1.006	0.217
F233	1.000	1.000	1.000	0.544
F234	0.081	0.002	2.096	0.145
F235	36.143	0.208	6.51E+04	0.247
F236	0.852	0.647	1.087	0.219
F237	62.235	0.016	3.19E+05	0.330
F238	0.993	0.981	1.003	0.216
F239	0.995	0.986	1.003	0.223
F240	1.003	0.350	2.665	0.994
F241	2.56E+05	0.000	1.05E+16	0.305
F242	1.022	0.955	1.102	0.538
F243	1.000	1.000	1.000	0.054
F244	0.987	0.966	1.006	0.188
F245	0.990	0.974	1.004	0.168
F246	1.000	1.000	1.000	0.054
F247	1.001	1.000	1.002	0.174
F248	0.993	0.980	1.004	0.211
F249	0.994	0.986	1.002	0.181
F250	0.999	0.998	1.001	0.315
F251	0.522	0.157	1.618	0.269
F252	1.000	1.000	1.001	0.213
F253	1.000	1.000	1.000	0.232
F254	1.005	0.998	1.014	0.183
F255	1.066	0.966	1.185	0.206
F256	1.011	0.964	1.064	0.651
F257	0.000	0.000	1.31E+12	0.379
F258	0.987	0.965	1.006	0.231
F259	0.000	0.000	1.10E+12	0.381
F260	1.97E+06	0.000	1.12E+18	0.280
F261	129.202	0.001	2.69E+07	0.425
F262	1.000	1.000	1.001	0.010
F263	7.850	0.078	863.214	0.378
F264	1.000	1.000	1.000	0.390
F265	1.000	1.000	1.000	0.015
F266	0.000	0.000	2.00E+05	0.299
F267	1.000	1.000	1.000	0.371
F268	0.000	0.000	1.04E+04	0.314
F269	0.000	0.000	7.24E+11	0.387
F270	0.490	0.106	2.072	0.341
F271	1.000	1.000	1.000	0.384
F272	0.002	0.000	173.391	0.279
F273	0.993	0.975	1.007	0.357
F274	1.000	1.000	1.000	0.329
F275	2.47E+10	0.000	3.00E+32	0.337
F276	0.001	0.000	4.383	0.142
F277	1.000	1.000	1.000	0.024
F278	1.001	1.000	1.002	0.017

F279	1.000	1.000	1.000	0.329
F280	1.000	1.000	1.001	0.482
F281	0.052	0.001	1.914	0.120
F282	1.125	0.933	1.577	0.326
F283	1.000	1.000	1.000	0.409
F284	1.000	1.000	1.000	0.380
F285	0.000	0.000	11.851	0.147
F286	0.000	0.000	3.83E+13	0.363
F287	1.909	0.489	12.267	0.409
F288	0.000	0.000	3.35E+19	0.348
F289	0.000	0.000	5.83E+03	0.370
F290	1.000	1.000	1.000	0.322
F291	0.937	0.798	1.049	0.341
F292	0.018	0.000	0.862	0.100
F293	1.360	0.944	2.024	0.107
F294	0.961	0.907	1.007	0.131
F295	1.000	0.998	1.001	0.583
F296	1.476	0.370	10.156	0.614
F297	1.000	1.000	1.000	0.021
F298	1.000	1.000	1.000	0.010
F299	0.011	0.000	1.311	0.078
F300	0.998	0.995	1.001	0.326
F301	0.001	0.000	2.507	0.104
F302	1.016	0.960	1.074	0.578
F303	4.78E+03	0.048	3.43E+09	0.172
F304	1.021	0.849	1.221	0.816
F305	1.000	1.000	1.000	0.553
F306	0.000	0.000	3.40E+22	0.450
F307	0.000	0.000	6.12E+24	0.664
F308	0.978	0.913	1.044	0.514
F309	0.989	0.956	1.022	0.514
F310	0.895	0.533	1.490	0.667
F311	1.031	1.009	1.061	0.018
F312	0.005	0.000	3.65E+07	0.659
F313	3.31E+16	0.001	1.45E+40	0.132
F314	0.000	0.000	1.29E+17	0.714
F315	0.981	0.954	1.004	0.146
F316	0.553	0.166	1.740	0.318
F317	25.314	0.015	5.04E+04	0.393
F318	0.947	0.879	1.006	0.115
F319	8.72E+04	0.001	2.54E+13	0.231
F320	8.993	0.011	8.26E+03	0.521
F321	1.180	0.000	2.98E+07	0.985
F322	1.000	0.998	1.001	0.605
F323	0.603	0.197	1.755	0.358
F324	0.025	0.000	2.868	0.154
F325	0.961	0.905	1.008	0.140

F326	1.000	1.000	1.000	0.175
F327	0.018	0.000	0.647	0.043
F328	2.57E+04	1.007	5.08E+11	0.128
F329	0.784	0.527	1.119	0.200
F330	16.701	0.011	3.09E+04	0.454
F331	0.979	0.949	1.004	0.134
F332	0.994	0.982	1.005	0.296
F333	34.371	2.646	882.202	0.015
F334	135.769	0.000	2.63E+09	0.560
F335	1.145	0.936	1.475	0.242
F336	1.000	1.000	1.000	0.064
F337	0.984	0.955	1.012	0.272
F338	0.986	0.963	1.006	0.183
F339	1.000	1.000	1.000	0.074
F340	1.000	0.998	1.003	0.786
F341	0.986	0.967	1.003	0.127
F342	0.994	0.981	1.005	0.298
F343	1.001	0.998	1.004	0.458
F344	0.537	0.166	1.635	0.281
F345	1.000	0.998	1.001	0.799
F346	1.000	1.000	1.000	0.131
F347	1.010	0.997	1.025	0.138
F348	0.945	0.751	1.170	0.610
F349	1.239	1.001	1.600	0.069
F350	0.000	0.000	3.78E+23	0.636
F351	0.960	0.907	1.007	0.125
F352	0.000	0.000	5.25E+23	0.661
F353	721.046	0.000	1.33E+11	0.488
F354	10.204	0.003	3.37E+04	0.565
F355	1.000	1.000	1.000	0.010
F356	3.006	0.122	76.661	0.495
F357	1.000	0.998	1.001	0.578
F358	1.000	1.000	1.000	0.015
F359	0.000	0.000	4.43E+04	0.356
F360	1.000	0.999	1.001	0.576
F361	0.002	0.000	3.74E+03	0.388
F362	0.000	0.000	2.66E+24	0.792
F363	0.473	0.097	2.097	0.331
F364	1.000	0.998	1.001	0.583
F365	0.009	0.000	102.929	0.322
F366	0.968	0.925	1.006	0.120
F367	1.000	1.000	1.000	0.137
F368	5.33E+04	0.000	1.81E+18	0.453
F369	0.000	0.000	1.239	0.076
F370	1.000	1.000	1.000	0.026
F371	1.001	1.000	1.002	0.018
F372	1.000	1.000	1.000	0.137

F373	0.999	0.998	1.001	0.485
F374	0.027	0.000	1.234	0.077
F375	1.034	1.001	1.093	0.165
F376	1.000	1.000	1.000	0.116
F377	1.000	0.998	1.001	0.592
F378	0.000	0.000	2.335	0.083
F379	0.002	0.000	1.06E+21	0.826
F380	1.132	0.351	4.295	0.837
F381	0.000	0.000	2.82E+22	0.774
F382	0.000	0.000	7.07E+03	0.354
F383	1.000	1.000	1.000	0.848
F384	0.741	0.443	1.044	0.152
F385	0.000	0.000	0.491	0.051
F386	1.240	1.069	1.501	0.011
F387	0.978	0.952	0.999	0.069
F388	1.000	0.999	1.000	0.922
F389	0.860	0.266	2.835	0.790
F390	1.000	1.000	1.000	0.022
F391	1.000	1.000	1.000	0.011
F392	0.005	0.000	0.408	0.033
F393	0.999	0.997	1.001	0.389
F394	0.000	0.000	0.346	0.061
F395	1.027	0.983	1.077	0.245
F396	841.826	0.000	1.25E+14	0.599
F397	1.055	0.914	1.222	0.461
F398	1.000	1.000	1.000	0.991
F399	0.000	0.000	1.70E+15	0.308
F400	0.000	0.000	1.09E+17	0.296
F401	1.008	0.967	1.051	0.690
F402	1.004	0.984	1.025	0.690
F403	0.702	0.412	1.154	0.172
F404	1.000	0.999	1.000	0.531
F405	2.18E+04	0.000	4.33E+15	0.428
F406	5.36E+25	1.16E+03	1.74E+55	0.051
F407	3.57E+07	0.000	1.70E+30	0.482
F408	0.985	0.966	0.999	0.070
F409	0.372	0.122	1.023	0.065
F410	1.49E+03	1.125	4.39E+06	0.056
F411	0.975	0.942	1.002	0.096
F412	2.38E+08	3.731	4.99E+17	0.052
F413	458.922	0.708	5.99E+05	0.075
F414	0.004	0.000	4.204	0.131
F415	1.000	0.999	1.000	0.955
F416	0.419	0.159	1.009	0.062
F417	0.061	0.000	6.316	0.275
F418	0.973	0.942	0.997	0.058
F419	1.000	1.000	1.000	0.177

F420	0.041	0.002	0.723	0.036
F421	693.460	1.126	2.66E+07	0.137
F422	0.700	0.480	0.951	0.040
F423	1.34E+03	1.070	3.96E+06	0.059
F424	0.988	0.974	0.999	0.051
F425	0.991	0.980	0.999	0.057
F426	0.951	0.396	2.282	0.910
F427	8.53E+05	0.405	1.13E+13	0.080
F428	1.021	0.994	1.057	0.176
F429	1.000	1.000	1.000	0.072
F430	0.978	0.955	0.998	0.050
F431	0.984	0.968	0.998	0.035
F432	1.000	1.000	1.000	0.070
F433	1.000	0.998	1.002	0.836
F434	0.989	0.977	0.999	0.037
F435	0.989	0.976	0.999	0.067
F436	1.000	0.998	1.001	0.749
F437	0.360	0.129	0.904	0.037
F438	1.000	0.999	1.001	0.937
F439	1.000	1.000	1.000	0.069
F440	1.005	1.000	1.012	0.067
F441	1.096	0.998	1.220	0.068
F442	1.010	0.988	1.034	0.411
F443	0.000	0.000	3.77E+16	0.292
F444	0.979	0.954	0.999	0.067
F445	0.000	0.000	8.77E+16	0.297
F446	2.74E+07	1.446	4.39E+15	0.057
F447	47.827	0.121	3.98E+04	0.220
F448	1.000	1.000	1.000	0.011
F449	5.942	0.505	96.935	0.174
F450	1.000	0.999	1.000	0.905
F451	1.000	1.000	1.000	0.017
F452	0.000	0.000	8.417	0.100
F453	1.000	1.000	1.000	0.976
F454	0.000	0.000	7.552	0.114
F455	0.000	0.000	5.75E+18	0.325
F456	0.258	0.066	0.888	0.039
F457	1.000	0.999	1.000	0.923
F458	0.001	0.000	2.025	0.085
F459	0.987	0.971	1.001	0.092
F460	1.000	1.000	1.000	0.051
F461	3.35E+16	590.984	1.76E+32	0.026
F462	0.000	0.000	0.451	0.055
F463	1.000	1.000	1.000	0.032
F464	1.001	1.000	1.003	0.024
F465	1.000	1.000	1.000	0.051
F466	1.000	0.999	1.001	0.835

F467	0.011	0.000	0.435	0.028
F468	1.027	1.002	1.068	0.105
F469	1.000	1.000	1.000	0.050
F470	1.000	0.999	1.000	0.939
F471	0.000	0.000	0.293	0.048
F472	0.000	0.000	3.11E+17	0.336
F473	0.786	0.230	2.664	0.685
F474	0.000	0.000	5.00E+18	0.344
F475	0.000	0.000	3.10E+03	0.346
F476	1.000	1.000	1.000	0.679
F477	0.866	0.670	1.000	0.158
F478	0.005	0.000	0.689	0.097
F479	1.475	1.097	2.132	0.019
F480	0.944	0.868	1.013	0.135
F481	1.001	0.999	1.002	0.373
F482	0.990	0.227	4.990	0.989
F483	1.000	1.000	1.000	0.027
F484	1.000	1.000	1.000	0.011
F485	0.006	0.000	0.687	0.053
F486	0.999	0.995	1.004	0.811
F487	0.001	0.000	1.189	0.092
F488	1.016	0.988	1.047	0.274
F489	5.375	0.020	1.87E+03	0.542
F490	1.040	0.938	1.155	0.451
F491	1.000	1.000	1.000	0.417
F492	0.000	0.000	3.43E+10	0.294
F493	0.000	0.000	1.04E+09	0.276
F494	1.030	0.964	1.104	0.377
F495	1.015	0.982	1.051	0.377
F496	0.753	0.451	1.233	0.263
F497	1.004	0.995	1.016	0.411
F498	411.546	0.000	2.22E+09	0.434
F499	1.81E+33	2.64E+08	8.53E+64	0.022
F500	2.27E+05	0.000	3.52E+18	0.402
F501	0.968	0.926	1.005	0.112
F502	0.429	0.133	1.289	0.140
F503	163.343	0.388	9.80E+04	0.105
F504	0.947	0.858	1.028	0.227
F505	6.52E+10	710.691	1.62E+20	0.014
F506	46.693	0.274	1.03E+04	0.148
F507	0.017	0.000	3.32E+05	0.634
F508	1.001	0.999	1.002	0.359
F509	0.483	0.169	1.306	0.159
F510	1.799	0.026	115.243	0.777
F511	0.934	0.854	1.008	0.105
F512	1.000	1.000	1.000	0.372
F513	0.064	0.001	1.597	0.119

F514	444.679	0.255	5.82E+08	0.255
F515	0.663	0.407	1.033	0.081
F516	110.528	0.266	6.34E+04	0.132
F517	0.965	0.922	1.003	0.099
F518	0.988	0.973	1.002	0.101
F519	1.762	0.359	9.412	0.488
F520	3.29E+03	0.060	4.24E+08	0.155
F521	0.960	0.775	1.152	0.669
F522	1.000	1.000	1.000	0.047
F523	0.971	0.935	1.004	0.097
F524	0.977	0.951	1.002	0.086
F525	1.000	1.000	1.000	0.053
F526	1.003	1.000	1.006	0.030
F527	0.982	0.961	1.002	0.098
F528	0.988	0.973	1.003	0.127
F529	0.999	0.996	1.001	0.382
F530	0.426	0.141	1.190	0.113
F531	1.001	1.000	1.003	0.092
F532	1.000	1.000	1.000	0.136
F533	1.014	0.999	1.031	0.071
F534	1.078	0.966	1.215	0.186
F535	1.115	0.929	1.443	0.314
F536	0.000	0.000	3.72E+08	0.272
F537	0.946	0.872	1.013	0.140
F538	0.000	0.000	9.99E+08	0.277
F539	4.53E+04	0.081	8.15E+10	0.123
F540	9.180	0.166	660.889	0.283
F541	1.000	1.000	1.000	0.011
F542	2.806	0.526	16.993	0.234
F543	1.001	0.999	1.002	0.403
F544	1.000	1.000	1.000	0.019
F545	0.000	0.000	25.167	0.150
F546	1.000	1.000	1.001	0.315
F547	0.001	0.000	15.125	0.172
F548	0.000	0.000	2.05E+11	0.340
F549	0.284	0.048	1.514	0.150
F550	1.001	0.999	1.002	0.373
F551	0.006	0.000	4.129	0.133
F552	0.980	0.932	1.026	0.405
F553	1.000	1.000	1.000	0.069
F554	2.29E+09	0.036	1.52E+21	0.099
F555	0.001	0.000	36.736	0.259
F556	1.000	1.000	1.000	0.036
F557	1.001	1.000	1.002	0.021
F558	1.000	1.000	1.000	0.069
F559	1.001	0.999	1.003	0.427
F560	0.012	0.000	0.646	0.046

F561	1.008	1.001	1.020	0.097
F562	1.000	1.000	1.000	0.037
F563	1.001	0.999	1.002	0.378
F564	0.000	0.000	185.791	0.250
F565	0.000	0.000	1.25E+10	0.323
F566	0.871	0.254	3.071	0.822
F567	0.000	0.000	6.86E+13	0.348
F568	0.000	0.000	3.12E+04	0.338
F569	1.000	1.000	1.001	0.357
F570	0.839	0.535	1.134	0.330
F571	0.000	0.000	0.119	0.054
F572	1.140	1.016	1.307	0.037
F573	0.958	0.872	1.043	0.340
F574	1.001	0.998	1.005	0.495
F575	1.386	0.424	6.143	0.613
F576	1.000	1.000	1.000	0.023
F577	1.000	1.000	1.000	0.012
F578	0.024	0.000	2.270	0.143
F579	0.999	0.989	1.008	0.796
F580	0.006	0.000	6.202	0.224
F581	1.010	0.984	1.038	0.454
F582	1.821	0.436	8.287	0.406
F583	1.032	0.927	1.148	0.561
F584	1.000	1.000	1.000	0.312
F585	0.000	0.000	1.49E+10	0.361
F586	0.001	0.000	8.84E+08	0.630
F587	1.029	0.928	1.144	0.585
F588	1.014	0.963	1.070	0.585
F589	0.900	0.597	1.346	0.609
F590	0.875	0.706	1.030	0.151
F591	3.121	0.000	1.27E+06	0.862
F592	1.95E+18	0.132	5.15E+42	0.105
F593	47.384	0.000	8.03E+11	0.746
F594	0.978	0.935	1.019	0.315
F595	0.720	0.283	1.779	0.480
F596	8.285	0.045	1.78E+03	0.430
F597	0.942	0.831	1.055	0.319
F598	6.67E+06	0.094	5.69E+15	0.109
F599	5.448	0.064	513.281	0.456
F600	1.16E+05	0.000	6.44E+29	0.520
F601	1.001	0.998	1.005	0.461
F602	0.773	0.335	1.745	0.537
F603	0.066	0.000	5.088	0.267
F604	0.956	0.870	1.041	0.323
F605	1.000	0.999	1.000	0.296
F606	0.017	0.000	0.822	0.067
F607	4.65E+06	0.338	2.41E+20	0.228

F608	0.822	0.533	1.237	0.357
F609	7.912	0.042	1.69E+03	0.440
F610	0.977	0.931	1.022	0.331
F611	0.995	0.981	1.008	0.450
F612	0.398	0.006	12.477	0.617
F613	14.091	0.003	8.71E+04	0.546
F614	1.260	0.785	2.289	0.380
F615	1.000	1.000	1.000	0.057
F616	0.987	0.954	1.019	0.430
F617	0.990	0.965	1.013	0.396
F618	1.000	1.000	1.000	0.093
F619	1.002	0.998	1.007	0.313
F620	0.991	0.971	1.010	0.370
F621	0.994	0.978	1.008	0.391
F622	0.999	0.995	1.003	0.584
F623	0.733	0.308	1.698	0.471
F624	1.001	0.999	1.003	0.422
F625	1.000	1.000	1.000	0.340
F626	1.006	0.992	1.022	0.420
F627	1.024	0.741	1.404	0.885
F628	0.934	0.439	1.948	0.849
F629	0.000	0.000	5.29E+08	0.592
F630	0.959	0.874	1.042	0.340
F631	0.001	0.000	4.70E+08	0.636
F632	25.193	0.001	6.77E+05	0.528
F633	4.278	0.090	216.962	0.456
F634	1.000	1.000	1.000	0.012
F635	1.907	0.397	9.482	0.417
F636	1.001	0.998	1.005	0.539
F637	1.000	1.000	1.000	0.018
F638	0.003	0.000	364.343	0.337
F639	1.001	0.999	1.004	0.372
F640	0.018	0.000	88.908	0.354
F641	0.157	0.000	1.65E+08	0.863
F642	0.750	0.206	2.663	0.656
F643	1.001	0.998	1.005	0.496
F644	0.049	0.000	17.000	0.316
F645	0.981	0.917	1.045	0.553
F646	1.000	1.000	1.000	0.092
F647	76.765	0.000	1.45E+08	0.551
F648	0.043	0.000	353.283	0.527
F649	1.000	1.000	1.000	0.031
F650	1.001	1.000	1.002	0.019
F651	1.000	1.000	1.000	0.092
F652	1.001	0.997	1.006	0.517
F653	0.037	0.000	1.664	0.116
F654	1.002	1.000	1.004	0.199

F655	1.000	1.000	1.000	0.043
F656	1.001	0.998	1.005	0.508
F657	0.033	0.000	1.38E+04	0.616
F658	0.135	0.000	1.57E+06	0.814
F659	1.058	0.494	2.308	0.885
F660	0.043	0.000	5.30E+07	0.776
F661	0.000	0.000	1.36E+04	0.350
F662	1.000	0.999	1.001	0.881
F663	0.533	0.146	1.307	0.233
F664	0.000	0.000	2.002	0.087
F665	1.073	1.020	1.147	0.017
F666	0.975	0.919	1.027	0.358
F667	1.002	1.000	1.003	0.064
F668	1.492	0.415	8.591	0.578
F669	1.000	1.000	1.000	0.025
F670	1.000	1.000	1.000	0.011
F671	0.050	0.000	3.065	0.178
F672	1.002	0.999	1.007	0.217
F673	0.010	0.000	6.227	0.213
F674	1.014	0.973	1.057	0.498
F675	0.573	0.002	83.126	0.829
F676	1.034	0.893	1.196	0.648
F677	1.000	1.000	1.000	0.075
F678	0.000	0.000	47.400	0.219
F679	0.000	0.000	6.95E+04	0.251
F680	1.076	1.003	1.167	0.055
F681	1.037	1.001	1.080	0.055
F682	0.921	0.597	1.414	0.704
F683	0.997	0.968	1.026	0.818
F684	0.186	0.000	2.38E+07	0.861
F685	6.48E+26	2.98E+05	5.82E+51	0.023
F686	0.376	0.000	1.23E+16	0.960
F687	0.987	0.960	1.012	0.338
F688	0.752	0.285	1.941	0.556
F689	5.345	0.015	2.04E+03	0.574
F690	0.967	0.896	1.036	0.358
F691	1.48E+09	30.516	5.39E+17	0.026
F692	5.121	0.023	1.20E+03	0.551
F693	0.000	0.000	561.370	0.291
F694	1.002	1.000	1.004	0.060
F695	0.754	0.304	1.840	0.536
F696	7.051	0.193	307.711	0.289
F697	0.972	0.916	1.025	0.312
F698	1.000	1.000	1.000	0.412
F699	0.065	0.002	1.491	0.109
F700	966.807	0.127	5.87E+09	0.264
F701	0.860	0.603	1.200	0.385

F702	7.403	0.018	3.38E+03	0.514
F703	0.984	0.954	1.012	0.286
F704	0.996	0.985	1.007	0.460
F705	0.900	0.072	11.301	0.933
F706	38.659	0.000	1.05E+07	0.561
F707	0.879	0.654	1.111	0.333
F708	1.000	1.000	1.000	0.052
F709	0.989	0.962	1.016	0.435
F710	0.991	0.971	1.011	0.391
F711	1.000	1.000	1.000	0.075
F712	1.002	1.000	1.005	0.069
F713	0.993	0.975	1.009	0.383
F714	0.994	0.982	1.006	0.368
F715	0.997	0.994	1.000	0.057
F716	0.707	0.270	1.805	0.470
F717	1.001	1.000	1.003	0.055
F718	1.000	1.000	1.000	0.361
F719	1.005	0.993	1.018	0.384
F720	1.085	0.926	1.284	0.313
F721	0.906	0.641	1.247	0.549
F722	0.000	0.000	2.31E+04	0.254
F723	0.975	0.920	1.027	0.361
F724	0.000	0.000	6.13E+04	0.253
F725	86.111	0.000	1.42E+08	0.535
F726	8.421	0.023	3.33E+03	0.472
F727	1.000	1.000	1.000	0.011
F728	2.506	0.239	27.382	0.439
F729	1.002	1.000	1.004	0.067
F730	1.000	1.000	1.000	0.017
F731	0.001	0.000	4.64E+03	0.372
F732	1.001	1.000	1.003	0.053
F733	0.007	0.000	550.449	0.384
F734	0.000	0.000	2.68E+06	0.257
F735	0.713	0.183	2.719	0.620
F736	1.002	1.000	1.003	0.063
F737	0.030	0.000	48.199	0.352
F738	0.988	0.941	1.035	0.618
F739	1.000	1.000	1.000	0.149
F740	223.023	0.000	1.38E+12	0.634
F741	0.083	0.000	1.20E+03	0.632
F742	1.000	1.000	1.000	0.030
F743	1.001	1.000	1.002	0.018
F744	1.000	1.000	1.000	0.149
F745	1.002	1.000	1.005	0.070
F746	0.072	0.002	2.103	0.144
F747	1.005	0.995	1.017	0.346
F748	1.000	1.000	1.000	0.100

F749	1.002	1.000	1.003	0.063
F750	0.064	0.000	7.14E+04	0.707
F751	0.000	0.000	5.38E+04	0.242
F752	1.198	0.452	3.503	0.722
F753	0.000	0.000	1.72E+05	0.241
F754	0.000	0.000	1.18E+03	0.381
F755	1.000	1.000	1.000	0.158
F756	0.816	0.461	1.244	0.397
F757	0.000	0.000	0.964	0.064
F758	1.119	0.995	1.280	0.073
F759	0.996	0.990	1.000	0.130
F760	1.000	1.000	1.000	0.052
F761	1.023	0.331	3.389	0.969
F762	1.000	1.000	1.000	0.022
F763	1.000	1.000	1.000	0.011
F764	0.019	0.000	0.618	0.040
F765	1.000	1.000	1.000	0.444
F766	0.002	0.000	0.426	0.055
F767	1.044	0.982	1.117	0.184
F768	0.000	0.000	1.23E+09	0.149
F769	1.118	0.902	1.402	0.313
F770	1.000	1.000	1.000	0.027
F771	0.000	0.000	5.05E+03	0.279
F772	0.000	0.000	7.489	0.242
F773	1.015	1.000	1.031	0.052
F774	1.008	1.000	1.015	0.052
F775	0.805	0.485	1.278	0.368
F776	1.000	1.000	1.000	0.908
F777	2.05E+05	0.000	5.06E+25	0.593
F778	3.99E+24	58.577	1.38E+56	0.074
F779	2.20E+04	0.000	1.13E+43	0.817
F780	0.994	0.986	1.000	0.073
F781	0.459	0.171	1.101	0.096
F782	1.02E+03	1.017	2.61E+06	0.062
F783	0.992	0.978	1.003	0.209
F784	3.89E+08	14.277	5.50E+17	0.040
F785	595.342	0.782	1.06E+06	0.072
F786	0.688	0.019	19.955	0.830
F787	1.000	1.000	1.000	0.052
F788	0.555	0.228	1.179	0.153
F789	1.797	0.049	64.782	0.746
F790	0.996	0.989	1.000	0.130
F791	1.000	1.000	1.000	0.646
F792	0.119	0.006	2.001	0.147
F793	112.152	0.995	1.46E+05	0.118
F794	0.810	0.639	0.974	0.046
F795	1.04E+03	1.165	2.54E+06	0.058

F796	0.999	0.997	1.000	0.153
F797	0.993	0.985	0.998	0.047
F798	0.672	0.478	0.905	0.014
F799	2.85E+08	0.178	6.50E+18	0.086
F800	1.013	1.003	1.026	0.024
F801	1.000	1.000	1.000	0.089
F802	0.986	0.971	0.997	0.047
F803	0.993	0.985	0.999	0.057
F804	1.000	1.000	1.000	0.073
F805	1.000	1.000	1.001	0.305
F806	0.997	0.992	1.000	0.155
F807	1.003	1.000	1.009	0.147
F808	1.000	0.999	1.000	0.259
F809	0.447	0.173	1.010	0.070
F810	1.000	1.000	1.001	0.184
F811	1.000	1.000	1.000	0.130
F812	1.003	1.001	1.006	0.056
F813	1.019	0.998	1.043	0.088
F814	1.008	1.002	1.017	0.041
F815	0.000	0.000	45.526	0.245
F816	0.996	0.991	1.000	0.138
F817	0.000	0.000	33.674	0.243
F818	2.20E+09	0.275	2.81E+20	0.077
F819	634.195	0.133	9.72E+06	0.154
F820	1.000	1.000	1.001	0.012
F821	13.932	0.536	566.686	0.131
F822	1.000	1.000	1.000	0.059
F823	1.000	1.000	1.000	0.016
F824	0.000	0.000	7.564	0.093
F825	1.000	1.000	1.000	0.035
F826	0.000	0.000	5.799	0.100
F827	0.000	0.000	5.937	0.233
F828	0.391	0.118	1.092	0.094
F829	1.000	1.000	1.000	0.052
F830	0.000	0.000	1.981	0.084
F831	0.999	0.995	1.002	0.433
F832	1.000	1.000	1.000	0.088
F833	4.73E+17	0.000	3.83E+40	0.111
F834	0.001	0.000	0.410	0.036
F835	1.000	1.000	1.000	0.030
F836	1.002	1.000	1.004	0.028
F837	1.000	1.000	1.000	0.088
F838	1.000	1.000	1.000	0.092
F839	0.035	0.001	0.698	0.041
F840	1.267	0.930	2.103	0.233
F841	1.000	1.000	1.000	0.041
F842	1.000	1.000	1.000	0.049

F843	0.000	0.000	0.249	0.031
F844	0.000	0.000	6.46E+05	0.223
F845	1.670	0.528	6.402	0.411
F846	0.000	0.000	5.07E+06	0.233
F847	0.000	0.000	1.77E+04	0.288
F848	1.000	1.000	1.000	0.450
F849	0.962	0.900	1.008	0.193
F850	0.051	0.001	0.693	0.084
F851	5.762	1.002	39.129	0.057
