# Supplementary material for: Pretreatment Thoracic CT Radiomic Features to Predict Brain Metastases in Patients With ALK-Rearranged Non-Small Cell Lung Cancer
Source: Front Genet. 2022 Feb 25;13:772090. doi: 10.3389/fgene.2022.772090 (PMC8914538; doi:10.3389/fgene.2022.772090)
Supplement: Supplementary file 2 [file DataSheet3.pdf]

**Supplementary S1.** Description of the five radiomic features in the prediction model

1. **Original.GLCM.Contrast:** A Gray Level Co-Occurrence Matrix（**GLCM**） based texture feature.

**GLCM** is defined as $P(i,j;\delta,\alpha)$, a matrix with size $N_g \times N_g$ describing the second-order joint probability function of an image, where the $(i,j)$ th element represents the number of times the combination of intensity levels $i$ and $j$ occur in two pixels in the image, that are separated by a distance of $\delta$ pixels in direction $\alpha$, and $N_g$ is the number of discrete gray level intensities.

**Contrast** is calculated with the following formula:

$$contrast = \sum_{i=1}^{N_g}\sum_{j=1}^{N_g}|i-j|^2 P(i,j)$$

2. **Wavelet_LHH.GLCM.ClusterShade:** A Wavelet-based feature transformed from **GLCM** feature **Cluster shade**.

Wavelet transform effectively decouples textural information by decomposing the original image, in a similar manner as Fourier analysis, in low- and high- frequencies. In this study, a discrete, one-level and undecimated three dimensional wavelet transform was applied to each CT image, which decomposes the original image $X$ into eight decompositions. Consider $L$ and $H$ to be a low-pass (i.e. a scaling) and, respectively, a high-pass (i.e. a wavelet) function, and the wavelet decompositions of $X$ to be labeled as $X_{LLL}$, $X_{LLH}$, $X_{LHL}$, $X_{LHH}$, $X_{HLL}$, $X_{HLH}$, $X_{HHL}$ and $X_{HHH}$. For example, $X_{LLH}$ is then interpreted as the high-pass sub-band, resulting from directional filtering of $X$ with a low-pass filter along x-direction, a low-pass filter along y-direction and a high-pass filter along z-direction and is constructed as:

$$X_{LLH}(i,j,k) = \sum_{p=1}^{N_L}\sum_{q=1}^{N_L}\sum_{r=1}^{N_H} L(p)L(q)H(r)X(i+p,j+q,k+r)$$

Where $N_L$ is the length of filter $L$ and $N_H$ is the length of filter $H$. The other decompositions are constructed in a similar manner, applying their respective ordering of low or high-pass filtering in x, y and z-direction.

**Cluster shade** is calculated with the following formula:

$$cluster\ shade = \sum_{i=1}^{N_g}\sum_{j=1}^{N_g}\left[i + j - \mu_x(i) - \mu_y(j)\right]^3 P(i,j)$$

3. **Wavelet_LLH.GLSZM.SmallAreaEmphasis:** A Wavelet-based feature transformed from Gray Level Size Zone Matrix (**GLSZM**) feature **Small Area Emphasis (SAE).**

**GLSZM** quantifies size zone matrices in an image. Instead of looking in several directions as GLCM, it looks at flat zone size for the whole image. A flat zone is a group of connecting pixels with the same gray level. In a gray level size matrix $p(i,j)$, the $(i,j)$th element describes the frequency of matrices of size $j$ with gray level $i$, and $N_g$ is the number of discrete gray level intensities.

**SAE** is calculated with the following formula:

$$SAE = \frac{\sum_{i=1}^{N_g}\sum_{j=1}^{N_r}\left[\frac{p(i,j)}{j^2}\right]}{\sum_{i=1}^{N_g}\sum_{j=1}^{N_r}p(i,j)}$$

4. **Wavelet_HLH.Firstorder.Maximum:** A Wavelet-based feature transformed from **First-order** feature **Maximum.**

**First-order** features describe the distribution of voxel intensities within the CT image through commonly used and basic metrics. Let $X$ denote the three-dimensional image matrix. **Maximum** is the maximum intensity value of $X$.

5. **Wavelet_LLL.Firstorder.Skewness:** A Wavelet-based feature transformed from **First-order** feature **Skewness.**

**Skewness** is calculated with the following formula:

$$skewness = \frac{\frac{1}{N}\sum_{i=1}^{N}(X(i) - \bar{X})^3}{\left(\sqrt{\frac{1}{N}\sum_{i=1}^{N}(X(i) - \bar{X})^2}\right)^3}$$

**Reference:**


Coroller, T.P., Grossmann, P., Hou, Y., Rios Velazquez, E., Leijenaar, R.T., Hermann, G., et al. (2015). CT-based radiomic signature predicts distant metastasis in lung adenocarcinoma. *Radiother Oncol.* 114**,** 345-350. doi: 10.1016/j.radonc.2015.02.015
