# Supplementary material for: Pretreatment Thoracic CT Radiomic Features to Predict Brain Metastases in Patients With ALK-Rearranged Non-Small Cell Lung Cancer
Source: Front Genet. 2022 Feb 25;13:772090. doi: 10.3389/fgene.2022.772090 (PMC8914538; doi:10.3389/fgene.2022.772090)
Supplement: Supplementary file 3 [file DataSheet1.pdf]

Supplementary Table S1. Radiomic Features

Feature index	Image type	Feature Class	Feature Name
F1	original	shape	VoxelVolume
F2	original	shape	Maximum3DDiameter
F3	original	shape	MeshVolume
F4	original	shape	MajorAxisLength
F5	original	shape	Sphericity
F6	original	shape	LeastAxisLength
F7	original	shape	Elongation
F8	original	shape	SurfaceVolumeRatio
F9	original	shape	Maximum2DDiameterSlice
F10	original	shape	Flatness
F11	original	shape	SurfaceArea
F12	original	shape	MinorAxisLength
F13	original	shape	Maximum2DDiameterColumn
F14	original	shape	Maximum2DDiameterRow
F15	original	gldm	GrayLevelVariance
F16	original	gldm	HighGrayLevelEmphasis
F17	original	gldm	DependenceEntropy
F18	original	gldm	DependenceNonUniformity
F19	original	gldm	GrayLevelNonUniformity
F20	original	gldm	SmallDependenceEmphasis
F21	original	gldm	SmallDependenceHighGrayLevelEmphasis
F22	original	gldm	DependenceNonUniformityNormalized
F23	original	gldm	LargeDependenceEmphasis
F24	original	gldm	LargeDependenceLowGrayLevelEmphasis
F25	original	gldm	DependenceVariance
F26	original	gldm	LargeDependenceHighGrayLevelEmphasis
F27	original	gldm	SmallDependenceLowGrayLevelEmphasis
F28	original	gldm	LowGrayLevelEmphasis
F29	original	glcm	JointAverage
F30	original	glcm	SumAverage
F31	original	glcm	JointEntropy
F32	original	glcm	ClusterShade
F33	original	glcm	MaximumProbability
F34	original	glcm	Idmn
F35	original	glcm	JointEnergy
F36	original	glcm	Contrast
F37	original	glcm	DifferenceEntropy
F38	original	glcm	InverseVariance
F39	original	glcm	DifferenceVariance
F40	original	glcm	Idn
F41	original	glcm	Idm
F42	original	glcm	Correlation
F43	original	glcm	Autocorrelation
F44	original	glcm	SumEntropy
F45	original	glcm	MCC

F46	original	glcm	SumSquares
F47	original	glcm	ClusterProminence
F48	original	glcm	Imc2
F49	original	glcm	Imc1
F50	original	glcm	DifferenceAverage
F51	original	glcm	Id
F52	original	glcm	ClusterTendency
F53	original	firstorder	InterquartileRange
F54	original	firstorder	Skewness
F55	original	firstorder	Uniformity
F56	original	firstorder	Median
F57	original	firstorder	Energy
F58	original	firstorder	RobustMeanAbsoluteDeviation
F59	original	firstorder	MeanAbsoluteDeviation
F60	original	firstorder	TotalEnergy
F61	original	firstorder	Maximum
F62	original	firstorder	RootMeanSquared
F63	original	firstorder	90Percentile
F64	original	firstorder	Minimum
F65	original	firstorder	Entropy
F66	original	firstorder	Range
F67	original	firstorder	Variance
F68	original	firstorder	10Percentile
F69	original	firstorder	Kurtosis
F70	original	firstorder	Mean
F71	original	glrlm	ShortRunLowGrayLevelEmphasis
F72	original	glrlm	GrayLevelVariance
F73	original	glrlm	LowGrayLevelRunEmphasis
F74	original	glrlm	GrayLevelNonUniformityNormalized
F75	original	glrlm	RunVariance
F76	original	glrlm	GrayLevelNonUniformity
F77	original	glrlm	LongRunEmphasis
F78	original	glrlm	ShortRunHighGrayLevelEmphasis
F79	original	glrlm	RunLengthNonUniformity
F80	original	glrlm	ShortRunEmphasis
F81	original	glrlm	LongRunHighGrayLevelEmphasis
F82	original	glrlm	RunPercentage
F83	original	glrlm	LongRunLowGrayLevelEmphasis
F84	original	glrlm	RunEntropy
F85	original	glrlm	HighGrayLevelRunEmphasis
F86	original	glrlm	RunLengthNonUniformityNormalized
F87	original	glszm	GrayLevelVariance
F88	original	glszm	ZoneVariance
F89	original	glszm	GrayLevelNonUniformityNormalized
F90	original	glszm	SizeZoneNonUniformityNormalized
F91	original	glszm	SizeZoneNonUniformity
F92	original	glszm	GrayLevelNonUniformity

F93	original	glszm	LargeAreaEmphasis
F94	original	glszm	SmallAreaHighGrayLevelEmphasis
F95	original	glszm	ZonePercentage
F96	original	glszm	LargeAreaLowGrayLevelEmphasis
F97	original	glszm	LargeAreaHighGrayLevelEmphasis
F98	original	glszm	HighGrayLevelZoneEmphasis
F99	original	glszm	SmallAreaEmphasis
F100	original	glszm	LowGrayLevelZoneEmphasis
F101	original	glszm	ZoneEntropy
F102	original	glszm	SmallAreaLowGrayLevelEmphasis
F103	original	ngtdm	Coarseness
F104	original	ngtdm	Complexity
F105	original	ngtdm	Strength
F106	original	ngtdm	Contrast
F107	original	ngtdm	Busyness
F108	wavelet-HLL	gldm	GrayLevelVariance
F109	wavelet-HLL	gldm	HighGrayLevelEmphasis
F110	wavelet-HLL	gldm	DependenceEntropy
F111	wavelet-HLL	gldm	DependenceNonUniformity
F112	wavelet-HLL	gldm	GrayLevelNonUniformity
F113	wavelet-HLL	gldm	SmallDependenceEmphasis
F114	wavelet-HLL	gldm	SmallDependenceHighGrayLevelEmphasis
F115	wavelet-HLL	gldm	DependenceNonUniformityNormalized
F116	wavelet-HLL	gldm	LargeDependenceEmphasis
F117	wavelet-HLL	gldm	LargeDependenceLowGrayLevelEmphasis
F118	wavelet-HLL	gldm	DependenceVariance
F119	wavelet-HLL	gldm	LargeDependenceHighGrayLevelEmphasis
F120	wavelet-HLL	gldm	SmallDependenceLowGrayLevelEmphasis
F121	wavelet-HLL	gldm	LowGrayLevelEmphasis
F122	wavelet-HLL	glcm	JointAverage
F123	wavelet-HLL	glcm	SumAverage
F124	wavelet-HLL	glcm	JointEntropy
F125	wavelet-HLL	glcm	ClusterShade
F126	wavelet-HLL	glcm	MaximumProbability
F127	wavelet-HLL	glcm	Idmn
F128	wavelet-HLL	glcm	JointEnergy
F129	wavelet-HLL	glcm	Contrast
F130	wavelet-HLL	glcm	DifferenceEntropy
F131	wavelet-HLL	glcm	InverseVariance
F132	wavelet-HLL	glcm	DifferenceVariance
F133	wavelet-HLL	glcm	Idn
F134	wavelet-HLL	glcm	Idm
F135	wavelet-HLL	glcm	Correlation
F136	wavelet-HLL	glcm	Autocorrelation
F137	wavelet-HLL	glcm	SumEntropy
F138	wavelet-HLL	glcm	MCC
F139	wavelet-HLL	glcm	SumSquares

F140	wavelet-HLL	glcm	ClusterProminence
F141	wavelet-HLL	glcm	Imc2
F142	wavelet-HLL	glcm	Imc1
F143	wavelet-HLL	glcm	DifferenceAverage
F144	wavelet-HLL	glcm	Id
F145	wavelet-HLL	glcm	ClusterTendency
F146	wavelet-HLL	firstorder	InterquartileRange
F147	wavelet-HLL	firstorder	Skewness
F148	wavelet-HLL	firstorder	Uniformity
F149	wavelet-HLL	firstorder	Median
F150	wavelet-HLL	firstorder	Energy
F151	wavelet-HLL	firstorder	RobustMeanAbsoluteDeviation
F152	wavelet-HLL	firstorder	MeanAbsoluteDeviation
F153	wavelet-HLL	firstorder	TotalEnergy
F154	wavelet-HLL	firstorder	Maximum
F155	wavelet-HLL	firstorder	RootMeanSquared
F156	wavelet-HLL	firstorder	90Percentile
F157	wavelet-HLL	firstorder	Minimum
F158	wavelet-HLL	firstorder	Entropy
F159	wavelet-HLL	firstorder	Range
F160	wavelet-HLL	firstorder	Variance
F161	wavelet-HLL	firstorder	10Percentile
F162	wavelet-HLL	firstorder	Kurtosis
F163	wavelet-HLL	firstorder	Mean
F164	wavelet-HLL	glrlm	ShortRunLowGrayLevelEmphasis
F165	wavelet-HLL	glrlm	GrayLevelVariance
F166	wavelet-HLL	glrlm	LowGrayLevelRunEmphasis
F167	wavelet-HLL	glrlm	GrayLevelNonUniformityNormalized
F168	wavelet-HLL	glrlm	RunVariance
F169	wavelet-HLL	glrlm	GrayLevelNonUniformity
F170	wavelet-HLL	glrlm	LongRunEmphasis
F171	wavelet-HLL	glrlm	ShortRunHighGrayLevelEmphasis
F172	wavelet-HLL	glrlm	RunLengthNonUniformity
F173	wavelet-HLL	glrlm	ShortRunEmphasis
F174	wavelet-HLL	glrlm	LongRunHighGrayLevelEmphasis
F175	wavelet-HLL	glrlm	RunPercentage
F176	wavelet-HLL	glrlm	LongRunLowGrayLevelEmphasis
F177	wavelet-HLL	glrlm	RunEntropy
F178	wavelet-HLL	glrlm	HighGrayLevelRunEmphasis
F179	wavelet-HLL	glrlm	RunLengthNonUniformityNormalized
F180	wavelet-HLL	glszm	GrayLevelVariance
F181	wavelet-HLL	glszm	ZoneVariance
F182	wavelet-HLL	glszm	GrayLevelNonUniformityNormalized
F183	wavelet-HLL	glszm	SizeZoneNonUniformityNormalized
F184	wavelet-HLL	glszm	SizeZoneNonUniformity
F185	wavelet-HLL	glszm	GrayLevelNonUniformity
F186	wavelet-HLL	glszm	LargeAreaEmphasis

F187	wavelet-HLL	glszm	SmallAreaHighGrayLevelEmphasis
F188	wavelet-HLL	glszm	ZonePercentage
F189	wavelet-HLL	glszm	LargeAreaLowGrayLevelEmphasis
F190	wavelet-HLL	glszm	LargeAreaHighGrayLevelEmphasis
F191	wavelet-HLL	glszm	HighGrayLevelZoneEmphasis
F192	wavelet-HLL	glszm	SmallAreaEmphasis
F193	wavelet-HLL	glszm	LowGrayLevelZoneEmphasis
F194	wavelet-HLL	glszm	ZoneEntropy
F195	wavelet-HLL	glszm	SmallAreaLowGrayLevelEmphasis
F196	wavelet-HLL	ngtdm	Coarseness
F197	wavelet-HLL	ngtdm	Complexity
F198	wavelet-HLL	ngtdm	Strength
F199	wavelet-HLL	ngtdm	Contrast
F200	wavelet-HLL	ngtdm	Busyness
F201	wavelet-LHL	gldm	GrayLevelVariance
F202	wavelet-LHL	gldm	HighGrayLevelEmphasis
F203	wavelet-LHL	gldm	DependenceEntropy
F204	wavelet-LHL	gldm	DependenceNonUniformity
F205	wavelet-LHL	gldm	GrayLevelNonUniformity
F206	wavelet-LHL	gldm	SmallDependenceEmphasis
F207	wavelet-LHL	gldm	SmallDependenceHighGrayLevelEmphasis
F208	wavelet-LHL	gldm	DependenceNonUniformityNormalized
F209	wavelet-LHL	gldm	LargeDependenceEmphasis
F210	wavelet-LHL	gldm	LargeDependenceLowGrayLevelEmphasis
F211	wavelet-LHL	gldm	DependenceVariance
F212	wavelet-LHL	gldm	LargeDependenceHighGrayLevelEmphasis
F213	wavelet-LHL	gldm	SmallDependenceLowGrayLevelEmphasis
F214	wavelet-LHL	gldm	LowGrayLevelEmphasis
F215	wavelet-LHL	glcm	JointAverage
F216	wavelet-LHL	glcm	SumAverage
F217	wavelet-LHL	glcm	JointEntropy
F218	wavelet-LHL	glcm	ClusterShade
F219	wavelet-LHL	glcm	MaximumProbability
F220	wavelet-LHL	glcm	Idmn
F221	wavelet-LHL	glcm	JointEnergy
F222	wavelet-LHL	glcm	Contrast
F223	wavelet-LHL	glcm	DifferenceEntropy
F224	wavelet-LHL	glcm	InverseVariance
F225	wavelet-LHL	glcm	DifferenceVariance
F226	wavelet-LHL	glcm	Idn
F227	wavelet-LHL	glcm	Idm
F228	wavelet-LHL	glcm	Correlation
F229	wavelet-LHL	glcm	Autocorrelation
F230	wavelet-LHL	glcm	SumEntropy
F231	wavelet-LHL	glcm	MCC
F232	wavelet-LHL	glcm	SumSquares
F233	wavelet-LHL	glcm	ClusterProminence

F234	wavelet-LHL	glcm	Imc2
F235	wavelet-LHL	glcm	Imc1
F236	wavelet-LHL	glcm	DifferenceAverage
F237	wavelet-LHL	glcm	Id
F238	wavelet-LHL	glcm	ClusterTendency
F239	wavelet-LHL	firstorder	InterquartileRange
F240	wavelet-LHL	firstorder	Skewness
F241	wavelet-LHL	firstorder	Uniformity
F242	wavelet-LHL	firstorder	Median
F243	wavelet-LHL	firstorder	Energy
F244	wavelet-LHL	firstorder	RobustMeanAbsoluteDeviation
F245	wavelet-LHL	firstorder	MeanAbsoluteDeviation
F246	wavelet-LHL	firstorder	TotalEnergy
F247	wavelet-LHL	firstorder	Maximum
F248	wavelet-LHL	firstorder	RootMeanSquared
F249	wavelet-LHL	firstorder	90Percentile
F250	wavelet-LHL	firstorder	Minimum
F251	wavelet-LHL	firstorder	Entropy
F252	wavelet-LHL	firstorder	Range
F253	wavelet-LHL	firstorder	Variance
F254	wavelet-LHL	firstorder	10Percentile
F255	wavelet-LHL	firstorder	Kurtosis
F256	wavelet-LHL	firstorder	Mean
F257	wavelet-LHL	glrlm	ShortRunLowGrayLevelEmphasis
F258	wavelet-LHL	glrlm	GrayLevelVariance
F259	wavelet-LHL	glrlm	LowGrayLevelRunEmphasis
F260	wavelet-LHL	glrlm	GrayLevelNonUniformityNormalized
F261	wavelet-LHL	glrlm	RunVariance
F262	wavelet-LHL	glrlm	GrayLevelNonUniformity
F263	wavelet-LHL	glrlm	LongRunEmphasis
F264	wavelet-LHL	glrlm	ShortRunHighGrayLevelEmphasis
F265	wavelet-LHL	glrlm	RunLengthNonUniformity
F266	wavelet-LHL	glrlm	ShortRunEmphasis
F267	wavelet-LHL	glrlm	LongRunHighGrayLevelEmphasis
F268	wavelet-LHL	glrlm	RunPercentage
F269	wavelet-LHL	glrlm	LongRunLowGrayLevelEmphasis
F270	wavelet-LHL	glrlm	RunEntropy
F271	wavelet-LHL	glrlm	HighGrayLevelRunEmphasis
F272	wavelet-LHL	glrlm	RunLengthNonUniformityNormalized
F273	wavelet-LHL	glszm	GrayLevelVariance
F274	wavelet-LHL	glszm	ZoneVariance
F275	wavelet-LHL	glszm	GrayLevelNonUniformityNormalized
F276	wavelet-LHL	glszm	SizeZoneNonUniformityNormalized
F277	wavelet-LHL	glszm	SizeZoneNonUniformity
F278	wavelet-LHL	glszm	GrayLevelNonUniformity
F279	wavelet-LHL	glszm	LargeAreaEmphasis
F280	wavelet-LHL	glszm	SmallAreaHighGrayLevelEmphasis

F281	wavelet-LHL	glszm	ZonePercentage
F282	wavelet-LHL	glszm	LargeAreaLowGrayLevelEmphasis
F283	wavelet-LHL	glszm	LargeAreaHighGrayLevelEmphasis
F284	wavelet-LHL	glszm	HighGrayLevelZoneEmphasis
F285	wavelet-LHL	glszm	SmallAreaEmphasis
F286	wavelet-LHL	glszm	LowGrayLevelZoneEmphasis
F287	wavelet-LHL	glszm	ZoneEntropy
F288	wavelet-LHL	glszm	SmallAreaLowGrayLevelEmphasis
F289	wavelet-LHL	ngtdm	Coarseness
F290	wavelet-LHL	ngtdm	Complexity
F291	wavelet-LHL	ngtdm	Strength
F292	wavelet-LHL	ngtdm	Contrast
F293	wavelet-LHL	ngtdm	Busyness
F294	wavelet-LHH	gldm	GrayLevelVariance
F295	wavelet-LHH	gldm	HighGrayLevelEmphasis
F296	wavelet-LHH	gldm	DependenceEntropy
F297	wavelet-LHH	gldm	DependenceNonUniformity
F298	wavelet-LHH	gldm	GrayLevelNonUniformity
F299	wavelet-LHH	gldm	SmallDependenceEmphasis
F300	wavelet-LHH	gldm	SmallDependenceHighGrayLevelEmphasis
F301	wavelet-LHH	gldm	DependenceNonUniformityNormalized
F302	wavelet-LHH	gldm	LargeDependenceEmphasis
F303	wavelet-LHH	gldm	LargeDependenceLowGrayLevelEmphasis
F304	wavelet-LHH	gldm	DependenceVariance
F305	wavelet-LHH	gldm	LargeDependenceHighGrayLevelEmphasis
F306	wavelet-LHH	gldm	SmallDependenceLowGrayLevelEmphasis
F307	wavelet-LHH	gldm	LowGrayLevelEmphasis
F308	wavelet-LHH	glcm	JointAverage
F309	wavelet-LHH	glcm	SumAverage
F310	wavelet-LHH	glcm	JointEntropy
F311	wavelet-LHH	glcm	ClusterShade
F312	wavelet-LHH	glcm	MaximumProbability
F313	wavelet-LHH	glcm	Idmn
F314	wavelet-LHH	glcm	JointEnergy
F315	wavelet-LHH	glcm	Contrast
F316	wavelet-LHH	glcm	DifferenceEntropy
F317	wavelet-LHH	glcm	InverseVariance
F318	wavelet-LHH	glcm	DifferenceVariance
F319	wavelet-LHH	glcm	Idn
F320	wavelet-LHH	glcm	Idm
F321	wavelet-LHH	glcm	Correlation
F322	wavelet-LHH	glcm	Autocorrelation
F323	wavelet-LHH	glcm	SumEntropy
F324	wavelet-LHH	glcm	MCC
F325	wavelet-LHH	glcm	SumSquares
F326	wavelet-LHH	glcm	ClusterProminence
F327	wavelet-LHH	glcm	Imc2

F328	wavelet-LHH	glcm	Imc1
F329	wavelet-LHH	glcm	DifferenceAverage
F330	wavelet-LHH	glcm	Id
F331	wavelet-LHH	glcm	ClusterTendency
F332	wavelet-LHH	firstorder	InterquartileRange
F333	wavelet-LHH	firstorder	Skewness
F334	wavelet-LHH	firstorder	Uniformity
F335	wavelet-LHH	firstorder	Median
F336	wavelet-LHH	firstorder	Energy
F337	wavelet-LHH	firstorder	RobustMeanAbsoluteDeviation
F338	wavelet-LHH	firstorder	MeanAbsoluteDeviation
F339	wavelet-LHH	firstorder	TotalEnergy
F340	wavelet-LHH	firstorder	Maximum
F341	wavelet-LHH	firstorder	RootMeanSquared
F342	wavelet-LHH	firstorder	90Percentile
F343	wavelet-LHH	firstorder	Minimum
F344	wavelet-LHH	firstorder	Entropy
F345	wavelet-LHH	firstorder	Range
F346	wavelet-LHH	firstorder	Variance
F347	wavelet-LHH	firstorder	10Percentile
F348	wavelet-LHH	firstorder	Kurtosis
F349	wavelet-LHH	firstorder	Mean
F350	wavelet-LHH	glrlm	ShortRunLowGrayLevelEmphasis
F351	wavelet-LHH	glrlm	GrayLevelVariance
F352	wavelet-LHH	glrlm	LowGrayLevelRunEmphasis
F353	wavelet-LHH	glrlm	GrayLevelNonUniformityNormalized
F354	wavelet-LHH	glrlm	RunVariance
F355	wavelet-LHH	glrlm	GrayLevelNonUniformity
F356	wavelet-LHH	glrlm	LongRunEmphasis
F357	wavelet-LHH	glrlm	ShortRunHighGrayLevelEmphasis
F358	wavelet-LHH	glrlm	RunLengthNonUniformity
F359	wavelet-LHH	glrlm	ShortRunEmphasis
F360	wavelet-LHH	glrlm	LongRunHighGrayLevelEmphasis
F361	wavelet-LHH	glrlm	RunPercentage
F362	wavelet-LHH	glrlm	LongRunLowGrayLevelEmphasis
F363	wavelet-LHH	glrlm	RunEntropy
F364	wavelet-LHH	glrlm	HighGrayLevelRunEmphasis
F365	wavelet-LHH	glrlm	RunLengthNonUniformityNormalized
F366	wavelet-LHH	glszm	GrayLevelVariance
F367	wavelet-LHH	glszm	ZoneVariance
F368	wavelet-LHH	glszm	GrayLevelNonUniformityNormalized
F369	wavelet-LHH	glszm	SizeZoneNonUniformityNormalized
F370	wavelet-LHH	glszm	SizeZoneNonUniformity
F371	wavelet-LHH	glszm	GrayLevelNonUniformity
F372	wavelet-LHH	glszm	LargeAreaEmphasis
F373	wavelet-LHH	glszm	SmallAreaHighGrayLevelEmphasis
F374	wavelet-LHH	glszm	ZonePercentage

F375	wavelet-LHH	glszm	LargeAreaLowGrayLevelEmphasis
F376	wavelet-LHH	glszm	LargeAreaHighGrayLevelEmphasis
F377	wavelet-LHH	glszm	HighGrayLevelZoneEmphasis
F378	wavelet-LHH	glszm	SmallAreaEmphasis
F379	wavelet-LHH	glszm	LowGrayLevelZoneEmphasis
F380	wavelet-LHH	glszm	ZoneEntropy
F381	wavelet-LHH	glszm	SmallAreaLowGrayLevelEmphasis
F382	wavelet-LHH	ngtdm	Coarseness
F383	wavelet-LHH	ngtdm	Complexity
F384	wavelet-LHH	ngtdm	Strength
F385	wavelet-LHH	ngtdm	Contrast
F386	wavelet-LHH	ngtdm	Busyness
F387	wavelet-LLH	gldm	GrayLevelVariance
F388	wavelet-LLH	gldm	HighGrayLevelEmphasis
F389	wavelet-LLH	gldm	DependenceEntropy
F390	wavelet-LLH	gldm	DependenceNonUniformity
F391	wavelet-LLH	gldm	GrayLevelNonUniformity
F392	wavelet-LLH	gldm	SmallDependenceEmphasis
F393	wavelet-LLH	gldm	SmallDependenceHighGrayLevelEmphasis
F394	wavelet-LLH	gldm	DependenceNonUniformityNormalized
F395	wavelet-LLH	gldm	LargeDependenceEmphasis
F396	wavelet-LLH	gldm	LargeDependenceLowGrayLevelEmphasis
F397	wavelet-LLH	gldm	DependenceVariance
F398	wavelet-LLH	gldm	LargeDependenceHighGrayLevelEmphasis
F399	wavelet-LLH	gldm	SmallDependenceLowGrayLevelEmphasis
F400	wavelet-LLH	gldm	LowGrayLevelEmphasis
F401	wavelet-LLH	glcm	JointAverage
F402	wavelet-LLH	glcm	SumAverage
F403	wavelet-LLH	glcm	JointEntropy
F404	wavelet-LLH	glcm	ClusterShade
F405	wavelet-LLH	glcm	MaximumProbability
F406	wavelet-LLH	glcm	Idmn
F407	wavelet-LLH	glcm	JointEnergy
F408	wavelet-LLH	glcm	Contrast
F409	wavelet-LLH	glcm	DifferenceEntropy
F410	wavelet-LLH	glcm	InverseVariance
F411	wavelet-LLH	glcm	DifferenceVariance
F412	wavelet-LLH	glcm	Idn
F413	wavelet-LLH	glcm	Idm
F414	wavelet-LLH	glcm	Correlation
F415	wavelet-LLH	glcm	Autocorrelation
F416	wavelet-LLH	glcm	SumEntropy
F417	wavelet-LLH	glcm	MCC
F418	wavelet-LLH	glcm	SumSquares
F419	wavelet-LLH	glcm	ClusterProminence
F420	wavelet-LLH	glcm	Imc2
F421	wavelet-LLH	glcm	Imc1

F422	wavelet-LLH	glcm	DifferenceAverage
F423	wavelet-LLH	glcm	Id
F424	wavelet-LLH	glcm	ClusterTendency
F425	wavelet-LLH	firstorder	InterquartileRange
F426	wavelet-LLH	firstorder	Skewness
F427	wavelet-LLH	firstorder	Uniformity
F428	wavelet-LLH	firstorder	Median
F429	wavelet-LLH	firstorder	Energy
F430	wavelet-LLH	firstorder	RobustMeanAbsoluteDeviation
F431	wavelet-LLH	firstorder	MeanAbsoluteDeviation
F432	wavelet-LLH	firstorder	TotalEnergy
F433	wavelet-LLH	firstorder	Maximum
F434	wavelet-LLH	firstorder	RootMeanSquared
F435	wavelet-LLH	firstorder	90Percentile
F436	wavelet-LLH	firstorder	Minimum
F437	wavelet-LLH	firstorder	Entropy
F438	wavelet-LLH	firstorder	Range
F439	wavelet-LLH	firstorder	Variance
F440	wavelet-LLH	firstorder	10Percentile
F441	wavelet-LLH	firstorder	Kurtosis
F442	wavelet-LLH	firstorder	Mean
F443	wavelet-LLH	glrlm	ShortRunLowGrayLevelEmphasis
F444	wavelet-LLH	glrlm	GrayLevelVariance
F445	wavelet-LLH	glrlm	LowGrayLevelRunEmphasis
F446	wavelet-LLH	glrlm	GrayLevelNonUniformityNormalized
F447	wavelet-LLH	glrlm	RunVariance
F448	wavelet-LLH	glrlm	GrayLevelNonUniformity
F449	wavelet-LLH	glrlm	LongRunEmphasis
F450	wavelet-LLH	glrlm	ShortRunHighGrayLevelEmphasis
F451	wavelet-LLH	glrlm	RunLengthNonUniformity
F452	wavelet-LLH	glrlm	ShortRunEmphasis
F453	wavelet-LLH	glrlm	LongRunHighGrayLevelEmphasis
F454	wavelet-LLH	glrlm	RunPercentage
F455	wavelet-LLH	glrlm	LongRunLowGrayLevelEmphasis
F456	wavelet-LLH	glrlm	RunEntropy
F457	wavelet-LLH	glrlm	HighGrayLevelRunEmphasis
F458	wavelet-LLH	glrlm	RunLengthNonUniformityNormalized
F459	wavelet-LLH	glszm	GrayLevelVariance
F460	wavelet-LLH	glszm	ZoneVariance
F461	wavelet-LLH	glszm	GrayLevelNonUniformityNormalized
F462	wavelet-LLH	glszm	SizeZoneNonUniformityNormalized
F463	wavelet-LLH	glszm	SizeZoneNonUniformity
F464	wavelet-LLH	glszm	GrayLevelNonUniformity
F465	wavelet-LLH	glszm	LargeAreaEmphasis
F466	wavelet-LLH	glszm	SmallAreaHighGrayLevelEmphasis
F467	wavelet-LLH	glszm	ZonePercentage
F468	wavelet-LLH	glszm	LargeAreaLowGrayLevelEmphasis

F469	wavelet-LLH	glszm	LargeAreaHighGrayLevelEmphasis
F470	wavelet-LLH	glszm	HighGrayLevelZoneEmphasis
F471	wavelet-LLH	glszm	SmallAreaEmphasis
F472	wavelet-LLH	glszm	LowGrayLevelZoneEmphasis
F473	wavelet-LLH	glszm	ZoneEntropy
F474	wavelet-LLH	glszm	SmallAreaLowGrayLevelEmphasis
F475	wavelet-LLH	ngtdm	Coarseness
F476	wavelet-LLH	ngtdm	Complexity
F477	wavelet-LLH	ngtdm	Strength
F478	wavelet-LLH	ngtdm	Contrast
F479	wavelet-LLH	ngtdm	Busyness
F480	wavelet-HLH	gldm	GrayLevelVariance
F481	wavelet-HLH	gldm	HighGrayLevelEmphasis
F482	wavelet-HLH	gldm	DependenceEntropy
F483	wavelet-HLH	gldm	DependenceNonUniformity
F484	wavelet-HLH	gldm	GrayLevelNonUniformity
F485	wavelet-HLH	gldm	SmallDependenceEmphasis
F486	wavelet-HLH	gldm	SmallDependenceHighGrayLevelEmphasis
F487	wavelet-HLH	gldm	DependenceNonUniformityNormalized
F488	wavelet-HLH	gldm	LargeDependenceEmphasis
F489	wavelet-HLH	gldm	LargeDependenceLowGrayLevelEmphasis
F490	wavelet-HLH	gldm	DependenceVariance
F491	wavelet-HLH	gldm	LargeDependenceHighGrayLevelEmphasis
F492	wavelet-HLH	gldm	SmallDependenceLowGrayLevelEmphasis
F493	wavelet-HLH	gldm	LowGrayLevelEmphasis
F494	wavelet-HLH	glcm	JointAverage
F495	wavelet-HLH	glcm	SumAverage
F496	wavelet-HLH	glcm	JointEntropy
F497	wavelet-HLH	glcm	ClusterShade
F498	wavelet-HLH	glcm	MaximumProbability
F499	wavelet-HLH	glcm	Idmn
F500	wavelet-HLH	glcm	JointEnergy
F501	wavelet-HLH	glcm	Contrast
F502	wavelet-HLH	glcm	DifferenceEntropy
F503	wavelet-HLH	glcm	InverseVariance
F504	wavelet-HLH	glcm	DifferenceVariance
F505	wavelet-HLH	glcm	Idn
F506	wavelet-HLH	glcm	Idm
F507	wavelet-HLH	glcm	Correlation
F508	wavelet-HLH	glcm	Autocorrelation
F509	wavelet-HLH	glcm	SumEntropy
F510	wavelet-HLH	glcm	MCC
F511	wavelet-HLH	glcm	SumSquares
F512	wavelet-HLH	glcm	ClusterProminence
F513	wavelet-HLH	glcm	Imc2
F514	wavelet-HLH	glcm	Imc1
F515	wavelet-HLH	glcm	DifferenceAverage

F516	wavelet-HLH	glcm	Id
F517	wavelet-HLH	glcm	ClusterTendency
F518	wavelet-HLH	firstorder	InterquartileRange
F519	wavelet-HLH	firstorder	Skewness
F520	wavelet-HLH	firstorder	Uniformity
F521	wavelet-HLH	firstorder	Median
F522	wavelet-HLH	firstorder	Energy
F523	wavelet-HLH	firstorder	RobustMeanAbsoluteDeviation
F524	wavelet-HLH	firstorder	MeanAbsoluteDeviation
F525	wavelet-HLH	firstorder	TotalEnergy
F526	wavelet-HLH	firstorder	Maximum
F527	wavelet-HLH	firstorder	RootMeanSquared
F528	wavelet-HLH	firstorder	90Percentile
F529	wavelet-HLH	firstorder	Minimum
F530	wavelet-HLH	firstorder	Entropy
F531	wavelet-HLH	firstorder	Range
F532	wavelet-HLH	firstorder	Variance
F533	wavelet-HLH	firstorder	10Percentile
F534	wavelet-HLH	firstorder	Kurtosis
F535	wavelet-HLH	firstorder	Mean
F536	wavelet-HLH	glrlm	ShortRunLowGrayLevelEmphasis
F537	wavelet-HLH	glrlm	GrayLevelVariance
F538	wavelet-HLH	glrlm	LowGrayLevelRunEmphasis
F539	wavelet-HLH	glrlm	GrayLevelNonUniformityNormalized
F540	wavelet-HLH	glrlm	RunVariance
F541	wavelet-HLH	glrlm	GrayLevelNonUniformity
F542	wavelet-HLH	glrlm	LongRunEmphasis
F543	wavelet-HLH	glrlm	ShortRunHighGrayLevelEmphasis
F544	wavelet-HLH	glrlm	RunLengthNonUniformity
F545	wavelet-HLH	glrlm	ShortRunEmphasis
F546	wavelet-HLH	glrlm	LongRunHighGrayLevelEmphasis
F547	wavelet-HLH	glrlm	RunPercentage
F548	wavelet-HLH	glrlm	LongRunLowGrayLevelEmphasis
F549	wavelet-HLH	glrlm	RunEntropy
F550	wavelet-HLH	glrlm	HighGrayLevelRunEmphasis
F551	wavelet-HLH	glrlm	RunLengthNonUniformityNormalized
F552	wavelet-HLH	glszm	GrayLevelVariance
F553	wavelet-HLH	glszm	ZoneVariance
F554	wavelet-HLH	glszm	GrayLevelNonUniformityNormalized
F555	wavelet-HLH	glszm	SizeZoneNonUniformityNormalized
F556	wavelet-HLH	glszm	SizeZoneNonUniformity
F557	wavelet-HLH	glszm	GrayLevelNonUniformity
F558	wavelet-HLH	glszm	LargeAreaEmphasis
F559	wavelet-HLH	glszm	SmallAreaHighGrayLevelEmphasis
F560	wavelet-HLH	glszm	ZonePercentage
F561	wavelet-HLH	glszm	LargeAreaLowGrayLevelEmphasis
F562	wavelet-HLH	glszm	LargeAreaHighGrayLevelEmphasis

F563	wavelet-HLH	glszm	HighGrayLevelZoneEmphasis
F564	wavelet-HLH	glszm	SmallAreaEmphasis
F565	wavelet-HLH	glszm	LowGrayLevelZoneEmphasis
F566	wavelet-HLH	glszm	ZoneEntropy
F567	wavelet-HLH	glszm	SmallAreaLowGrayLevelEmphasis
F568	wavelet-HLH	ngtdm	Coarseness
F569	wavelet-HLH	ngtdm	Complexity
F570	wavelet-HLH	ngtdm	Strength
F571	wavelet-HLH	ngtdm	Contrast
F572	wavelet-HLH	ngtdm	Busyness
F573	wavelet-HHH	gldm	GrayLevelVariance
F574	wavelet-HHH	gldm	HighGrayLevelEmphasis
F575	wavelet-HHH	gldm	DependenceEntropy
F576	wavelet-HHH	gldm	DependenceNonUniformity
F577	wavelet-HHH	gldm	GrayLevelNonUniformity
F578	wavelet-HHH	gldm	SmallDependenceEmphasis
F579	wavelet-HHH	gldm	SmallDependenceHighGrayLevelEmphasis
F580	wavelet-HHH	gldm	DependenceNonUniformityNormalized
F581	wavelet-HHH	gldm	LargeDependenceEmphasis
F582	wavelet-HHH	gldm	LargeDependenceLowGrayLevelEmphasis
F583	wavelet-HHH	gldm	DependenceVariance
F584	wavelet-HHH	gldm	LargeDependenceHighGrayLevelEmphasis
F585	wavelet-HHH	gldm	SmallDependenceLowGrayLevelEmphasis
F586	wavelet-HHH	gldm	LowGrayLevelEmphasis
F587	wavelet-HHH	glcm	JointAverage
F588	wavelet-HHH	glcm	SumAverage
F589	wavelet-HHH	glcm	JointEntropy
F590	wavelet-HHH	glcm	ClusterShade
F591	wavelet-HHH	glcm	MaximumProbability
F592	wavelet-HHH	glcm	Idmn
F593	wavelet-HHH	glcm	JointEnergy
F594	wavelet-HHH	glcm	Contrast
F595	wavelet-HHH	glcm	DifferenceEntropy
F596	wavelet-HHH	glcm	InverseVariance
F597	wavelet-HHH	glcm	DifferenceVariance
F598	wavelet-HHH	glcm	Idn
F599	wavelet-HHH	glcm	Idm
F600	wavelet-HHH	glcm	Correlation
F601	wavelet-HHH	glcm	Autocorrelation
F602	wavelet-HHH	glcm	SumEntropy
F603	wavelet-HHH	glcm	MCC
F604	wavelet-HHH	glcm	SumSquares
F605	wavelet-HHH	glcm	ClusterProminence
F606	wavelet-HHH	glcm	Imc2
F607	wavelet-HHH	glcm	Imc1
F608	wavelet-HHH	glcm	DifferenceAverage
F609	wavelet-HHH	glcm	Id

F610	wavelet-HHH	glcm	ClusterTendency
F611	wavelet-HHH	firstorder	InterquartileRange
F612	wavelet-HHH	firstorder	Skewness
F613	wavelet-HHH	firstorder	Uniformity
F614	wavelet-HHH	firstorder	Median
F615	wavelet-HHH	firstorder	Energy
F616	wavelet-HHH	firstorder	RobustMeanAbsoluteDeviation
F617	wavelet-HHH	firstorder	MeanAbsoluteDeviation
F618	wavelet-HHH	firstorder	TotalEnergy
F619	wavelet-HHH	firstorder	Maximum
F620	wavelet-HHH	firstorder	RootMeanSquared
F621	wavelet-HHH	firstorder	90Percentile
F622	wavelet-HHH	firstorder	Minimum
F623	wavelet-HHH	firstorder	Entropy
F624	wavelet-HHH	firstorder	Range
F625	wavelet-HHH	firstorder	Variance
F626	wavelet-HHH	firstorder	10Percentile
F627	wavelet-HHH	firstorder	Kurtosis
F628	wavelet-HHH	firstorder	Mean
F629	wavelet-HHH	glrlm	ShortRunLowGrayLevelEmphasis
F630	wavelet-HHH	glrlm	GrayLevelVariance
F631	wavelet-HHH	glrlm	LowGrayLevelRunEmphasis
F632	wavelet-HHH	glrlm	GrayLevelNonUniformityNormalized
F633	wavelet-HHH	glrlm	RunVariance
F634	wavelet-HHH	glrlm	GrayLevelNonUniformity
F635	wavelet-HHH	glrlm	LongRunEmphasis
F636	wavelet-HHH	glrlm	ShortRunHighGrayLevelEmphasis
F637	wavelet-HHH	glrlm	RunLengthNonUniformity
F638	wavelet-HHH	glrlm	ShortRunEmphasis
F639	wavelet-HHH	glrlm	LongRunHighGrayLevelEmphasis
F640	wavelet-HHH	glrlm	RunPercentage
F641	wavelet-HHH	glrlm	LongRunLowGrayLevelEmphasis
F642	wavelet-HHH	glrlm	RunEntropy
F643	wavelet-HHH	glrlm	HighGrayLevelRunEmphasis
F644	wavelet-HHH	glrlm	RunLengthNonUniformityNormalized
F645	wavelet-HHH	glszm	GrayLevelVariance
F646	wavelet-HHH	glszm	ZoneVariance
F647	wavelet-HHH	glszm	GrayLevelNonUniformityNormalized
F648	wavelet-HHH	glszm	SizeZoneNonUniformityNormalized
F649	wavelet-HHH	glszm	SizeZoneNonUniformity
F650	wavelet-HHH	glszm	GrayLevelNonUniformity
F651	wavelet-HHH	glszm	LargeAreaEmphasis
F652	wavelet-HHH	glszm	SmallAreaHighGrayLevelEmphasis
F653	wavelet-HHH	glszm	ZonePercentage
F654	wavelet-HHH	glszm	LargeAreaLowGrayLevelEmphasis
F655	wavelet-HHH	glszm	LargeAreaHighGrayLevelEmphasis
F656	wavelet-HHH	glszm	HighGrayLevelZoneEmphasis

F657	wavelet-HHH	glszm	SmallAreaEmphasis
F658	wavelet-HHH	glszm	LowGrayLevelZoneEmphasis
F659	wavelet-HHH	glszm	ZoneEntropy
F660	wavelet-HHH	glszm	SmallAreaLowGrayLevelEmphasis
F661	wavelet-HHH	ngtdm	Coarseness
F662	wavelet-HHH	ngtdm	Complexity
F663	wavelet-HHH	ngtdm	Strength
F664	wavelet-HHH	ngtdm	Contrast
F665	wavelet-HHH	ngtdm	Busyness
F666	wavelet-HHL	gldm	GrayLevelVariance
F667	wavelet-HHL	gldm	HighGrayLevelEmphasis
F668	wavelet-HHL	gldm	DependenceEntropy
F669	wavelet-HHL	gldm	DependenceNonUniformity
F670	wavelet-HHL	gldm	GrayLevelNonUniformity
F671	wavelet-HHL	gldm	SmallDependenceEmphasis
F672	wavelet-HHL	gldm	SmallDependenceHighGrayLevelEmphasis
F673	wavelet-HHL	gldm	DependenceNonUniformityNormalized
F674	wavelet-HHL	gldm	LargeDependenceEmphasis
F675	wavelet-HHL	gldm	LargeDependenceLowGrayLevelEmphasis
F676	wavelet-HHL	gldm	DependenceVariance
F677	wavelet-HHL	gldm	LargeDependenceHighGrayLevelEmphasis
F678	wavelet-HHL	gldm	SmallDependenceLowGrayLevelEmphasis
F679	wavelet-HHL	gldm	LowGrayLevelEmphasis
F680	wavelet-HHL	glcm	JointAverage
F681	wavelet-HHL	glcm	SumAverage
F682	wavelet-HHL	glcm	JointEntropy
F683	wavelet-HHL	glcm	ClusterShade
F684	wavelet-HHL	glcm	MaximumProbability
F685	wavelet-HHL	glcm	Idmn
F686	wavelet-HHL	glcm	JointEnergy
F687	wavelet-HHL	glcm	Contrast
F688	wavelet-HHL	glcm	DifferenceEntropy
F689	wavelet-HHL	glcm	InverseVariance
F690	wavelet-HHL	glcm	DifferenceVariance
F691	wavelet-HHL	glcm	Idn
F692	wavelet-HHL	glcm	Idm
F693	wavelet-HHL	glcm	Correlation
F694	wavelet-HHL	glcm	Autocorrelation
F695	wavelet-HHL	glcm	SumEntropy
F696	wavelet-HHL	glcm	MCC
F697	wavelet-HHL	glcm	SumSquares
F698	wavelet-HHL	glcm	ClusterProminence
F699	wavelet-HHL	glcm	Imc2
F700	wavelet-HHL	glcm	Imc1
F701	wavelet-HHL	glcm	DifferenceAverage
F702	wavelet-HHL	glcm	Id
F703	wavelet-HHL	glcm	ClusterTendency

F704	wavelet-HHL	firstorder	InterquartileRange
F705	wavelet-HHL	firstorder	Skewness
F706	wavelet-HHL	firstorder	Uniformity
F707	wavelet-HHL	firstorder	Median
F708	wavelet-HHL	firstorder	Energy
F709	wavelet-HHL	firstorder	RobustMeanAbsoluteDeviation
F710	wavelet-HHL	firstorder	MeanAbsoluteDeviation
F711	wavelet-HHL	firstorder	TotalEnergy
F712	wavelet-HHL	firstorder	Maximum
F713	wavelet-HHL	firstorder	RootMeanSquared
F714	wavelet-HHL	firstorder	90Percentile
F715	wavelet-HHL	firstorder	Minimum
F716	wavelet-HHL	firstorder	Entropy
F717	wavelet-HHL	firstorder	Range
F718	wavelet-HHL	firstorder	Variance
F719	wavelet-HHL	firstorder	10Percentile
F720	wavelet-HHL	firstorder	Kurtosis
F721	wavelet-HHL	firstorder	Mean
F722	wavelet-HHL	glrlm	ShortRunLowGrayLevelEmphasis
F723	wavelet-HHL	glrlm	GrayLevelVariance
F724	wavelet-HHL	glrlm	LowGrayLevelRunEmphasis
F725	wavelet-HHL	glrlm	GrayLevelNonUniformityNormalized
F726	wavelet-HHL	glrlm	RunVariance
F727	wavelet-HHL	glrlm	GrayLevelNonUniformity
F728	wavelet-HHL	glrlm	LongRunEmphasis
F729	wavelet-HHL	glrlm	ShortRunHighGrayLevelEmphasis
F730	wavelet-HHL	glrlm	RunLengthNonUniformity
F731	wavelet-HHL	glrlm	ShortRunEmphasis
F732	wavelet-HHL	glrlm	LongRunHighGrayLevelEmphasis
F733	wavelet-HHL	glrlm	RunPercentage
F734	wavelet-HHL	glrlm	LongRunLowGrayLevelEmphasis
F735	wavelet-HHL	glrlm	RunEntropy
F736	wavelet-HHL	glrlm	HighGrayLevelRunEmphasis
F737	wavelet-HHL	glrlm	RunLengthNonUniformityNormalized
F738	wavelet-HHL	glszm	GrayLevelVariance
F739	wavelet-HHL	glszm	ZoneVariance
F740	wavelet-HHL	glszm	GrayLevelNonUniformityNormalized
F741	wavelet-HHL	glszm	SizeZoneNonUniformityNormalized
F742	wavelet-HHL	glszm	SizeZoneNonUniformity
F743	wavelet-HHL	glszm	GrayLevelNonUniformity
F744	wavelet-HHL	glszm	LargeAreaEmphasis
F745	wavelet-HHL	glszm	SmallAreaHighGrayLevelEmphasis
F746	wavelet-HHL	glszm	ZonePercentage
F747	wavelet-HHL	glszm	LargeAreaLowGrayLevelEmphasis
F748	wavelet-HHL	glszm	LargeAreaHighGrayLevelEmphasis
F749	wavelet-HHL	glszm	HighGrayLevelZoneEmphasis
F750	wavelet-HHL	glszm	SmallAreaEmphasis

F751	wavelet-HHL	glszm	LowGrayLevelZoneEmphasis
F752	wavelet-HHL	glszm	ZoneEntropy
F753	wavelet-HHL	glszm	SmallAreaLowGrayLevelEmphasis
F754	wavelet-HHL	ngtdm	Coarseness
F755	wavelet-HHL	ngtdm	Complexity
F756	wavelet-HHL	ngtdm	Strength
F757	wavelet-HHL	ngtdm	Contrast
F758	wavelet-HHL	ngtdm	Busyness
F759	wavelet-LLL	gldm	GrayLevelVariance
F760	wavelet-LLL	gldm	HighGrayLevelEmphasis
F761	wavelet-LLL	gldm	DependenceEntropy
F762	wavelet-LLL	gldm	DependenceNonUniformity
F763	wavelet-LLL	gldm	GrayLevelNonUniformity
F764	wavelet-LLL	gldm	SmallDependenceEmphasis
F765	wavelet-LLL	gldm	SmallDependenceHighGrayLevelEmphasis
F766	wavelet-LLL	gldm	DependenceNonUniformityNormalized
F767	wavelet-LLL	gldm	LargeDependenceEmphasis
F768	wavelet-LLL	gldm	LargeDependenceLowGrayLevelEmphasis
F769	wavelet-LLL	gldm	DependenceVariance
F770	wavelet-LLL	gldm	LargeDependenceHighGrayLevelEmphasis
F771	wavelet-LLL	gldm	SmallDependenceLowGrayLevelEmphasis
F772	wavelet-LLL	gldm	LowGrayLevelEmphasis
F773	wavelet-LLL	glcm	JointAverage
F774	wavelet-LLL	glcm	SumAverage
F775	wavelet-LLL	glcm	JointEntropy
F776	wavelet-LLL	glcm	ClusterShade
F777	wavelet-LLL	glcm	MaximumProbability
F778	wavelet-LLL	glcm	Idmn
F779	wavelet-LLL	glcm	JointEnergy
F780	wavelet-LLL	glcm	Contrast
F781	wavelet-LLL	glcm	DifferenceEntropy
F782	wavelet-LLL	glcm	InverseVariance
F783	wavelet-LLL	glcm	DifferenceVariance
F784	wavelet-LLL	glcm	Idn
F785	wavelet-LLL	glcm	Idm
F786	wavelet-LLL	glcm	Correlation
F787	wavelet-LLL	glcm	Autocorrelation
F788	wavelet-LLL	glcm	SumEntropy
F789	wavelet-LLL	glcm	MCC
F790	wavelet-LLL	glcm	SumSquares
F791	wavelet-LLL	glcm	ClusterProminence
F792	wavelet-LLL	glcm	Imc2
F793	wavelet-LLL	glcm	Imc1
F794	wavelet-LLL	glcm	DifferenceAverage
F795	wavelet-LLL	glcm	Id
F796	wavelet-LLL	glcm	ClusterTendency
F797	wavelet-LLL	firstorder	InterquartileRange

F798	wavelet-LLL	firstorder	Skewness
F799	wavelet-LLL	firstorder	Uniformity
F800	wavelet-LLL	firstorder	Median
F801	wavelet-LLL	firstorder	Energy
F802	wavelet-LLL	firstorder	RobustMeanAbsoluteDeviation
F803	wavelet-LLL	firstorder	MeanAbsoluteDeviation
F804	wavelet-LLL	firstorder	TotalEnergy
F805	wavelet-LLL	firstorder	Maximum
F806	wavelet-LLL	firstorder	RootMeanSquared
F807	wavelet-LLL	firstorder	90Percentile
F808	wavelet-LLL	firstorder	Minimum
F809	wavelet-LLL	firstorder	Entropy
F810	wavelet-LLL	firstorder	Range
F811	wavelet-LLL	firstorder	Variance
F812	wavelet-LLL	firstorder	10Percentile
F813	wavelet-LLL	firstorder	Kurtosis
F814	wavelet-LLL	firstorder	Mean
F815	wavelet-LLL	glrlm	ShortRunLowGrayLevelEmphasis
F816	wavelet-LLL	glrlm	GrayLevelVariance
F817	wavelet-LLL	glrlm	LowGrayLevelRunEmphasis
F818	wavelet-LLL	glrlm	GrayLevelNonUniformityNormalized
F819	wavelet-LLL	glrlm	RunVariance
F820	wavelet-LLL	glrlm	GrayLevelNonUniformity
F821	wavelet-LLL	glrlm	LongRunEmphasis
F822	wavelet-LLL	glrlm	ShortRunHighGrayLevelEmphasis
F823	wavelet-LLL	glrlm	RunLengthNonUniformity
F824	wavelet-LLL	glrlm	ShortRunEmphasis
F825	wavelet-LLL	glrlm	LongRunHighGrayLevelEmphasis
F826	wavelet-LLL	glrlm	RunPercentage
F827	wavelet-LLL	glrlm	LongRunLowGrayLevelEmphasis
F828	wavelet-LLL	glrlm	RunEntropy
F829	wavelet-LLL	glrlm	HighGrayLevelRunEmphasis
F830	wavelet-LLL	glrlm	RunLengthNonUniformityNormalized
F831	wavelet-LLL	glszm	GrayLevelVariance
F832	wavelet-LLL	glszm	ZoneVariance
F833	wavelet-LLL	glszm	GrayLevelNonUniformityNormalized
F834	wavelet-LLL	glszm	SizeZoneNonUniformityNormalized
F835	wavelet-LLL	glszm	SizeZoneNonUniformity
F836	wavelet-LLL	glszm	GrayLevelNonUniformity
F837	wavelet-LLL	glszm	LargeAreaEmphasis
F838	wavelet-LLL	glszm	SmallAreaHighGrayLevelEmphasis
F839	wavelet-LLL	glszm	ZonePercentage
F840	wavelet-LLL	glszm	LargeAreaLowGrayLevelEmphasis
F841	wavelet-LLL	glszm	LargeAreaHighGrayLevelEmphasis
F842	wavelet-LLL	glszm	HighGrayLevelZoneEmphasis
F843	wavelet-LLL	glszm	SmallAreaEmphasis
F844	wavelet-LLL	glszm	LowGrayLevelZoneEmphasis

F845	wavelet-LLL	glszm	ZoneEntropy
F846	wavelet-LLL	glszm	SmallAreaLowGrayLevelEmphasis
F847	wavelet-LLL	ngtdm	Coarseness
F848	wavelet-LLL	ngtdm	Complexity
F849	wavelet-LLL	ngtdm	Strength
F850	wavelet-LLL	ngtdm	Contrast
F851	wavelet-LLL	ngtdm	Busyness
